# A scoping review of mycetoma profile in Egypt: revisiting the global endemicity map

**DOI:** 10.1093/trstmh/trac085

**Published:** 2022-09-09

**Authors:** Sarah A Ahmed, Tamer A El-Sobky, Sybren de Hoog, Sherif M Zaki, Mohamed Taha

**Affiliations:** Center of Expertise in Mycology Radboudumc, Canisius Wilhelmina Hospital, Geert Grooteplein Zuid 10, 6525 GA, Nijmegen, The Netherlands; Foundation Atlas of Clinical Fungi, Hilversum, The Netherlands; Division of Pediatric Orthopaedics, Department of Orthopaedic Surgery, Faculty of Medicine, Ain Shams University, Abbassia, Cairo, Egypt; Center of Expertise in Mycology Radboudumc, Canisius Wilhelmina Hospital, Geert Grooteplein Zuid 10, 6525 GA, Nijmegen, The Netherlands; Foundation Atlas of Clinical Fungi, Hilversum, The Netherlands; Mycology Unit, Department of Microbiology, Faculty of Science, Ain Shams University, Cairo Governorate 4392001, Cairo, Egypt; Department of Microbiology, Zagazig University, Ash Sharqia Governorate 7120001, Zagazig, Egypt

**Keywords:** actinomycetoma, eumycetoma, neglected tropical diseases, Egypt, epidemiology, northern Africa

## Abstract

Mycetoma is a chronic infectious disease endemic in sub-Saharan Africa (SSA), India and parts of South and North America. The epidemiologic profile of the disease in Egypt, which neighbours SSA, has not been explored previously. Therefore we conducted a scoping review of the literature on mycetoma in Egypt. We searched the literature comprehensively on MEDLINE and Google Scholar using free-text words and Medical Subject Headings and terms. Both published and non-peer-reviewed (grey literature) articles were included. The initial search identified 133 reports. Of these, only eight were found to be relevant and were included in the study. The total number of mycetoma patients was 59, reported between 1949 and 2015. There was a predilection for eumycetoma (44 of 59) patients (75%), while actinomycetoma constituted 15 patients (25%). Six patients were female, 28 were male and 25 were unreported. Children and adolescents constituted 3 of 59 (5%), 52 (88%) were adults and age was not provided for 4 patients. Only four patients (7%) were non-autochthonous. The incidence of mycetoma in Egypt is higher than previously reported. Egypt is probably a low-endemic country. An accurate estimate of the prevalence and epidemiology of mycetoma necessitates further research collaboration.

## Introduction

Mycetoma is a chronic disease of arid climate zones that primarily affects the skin and subcutaneous tissues, leading to severe disfigurement and disabilities. Infections are introduced traumatically, mainly via the lower limbs, and prevail among people working barefoot, such as farmers and field workers.^1,2^ The disease has a unique pathology in that it has a very long incubation period, is characterized by painless subcutaneous swelling and leads to the development of sinuses that open to the skin surface, discharging the causative agent in the form of characteristic grains.^1^ Diagnosis is based on a combination of tools, including direct examination of grains from sinus discharge, histopathological examination of tissues, imaging (X-ray and/or magnetic resonance imaging [MRI]) to determine the extent of infection and culturing of grains to isolate and identify the aetiologic agent.^2–6^ Mismanaged, undiagnosed infections eventually spread to underlying muscles, tendons and bones, causing extensive disfigurement and disability, and may necessitate radical surgery or amputation.^1,7–9^

Mycetoma has been discussed in the medical literature since the mid-1800s, yet its distribution and true burden are unknown.^10,11^ In addition, diagnosis, management and prevention pose challenges both in and outside endemic regions.^11–13^ Therefore, in 2016, the World Health Organization (WHO) assembly decided to add mycetoma to the list of the neglected tropical diseases (NTDs).^14^ Mycetoma is endemic in tropical countries of the so-called mycetoma belt, located between 15°S latitude and 30°N latitude.^1,3^ The highest endemicity is observed in Sudan,^15^ Senegal^16^ and India,^17^ with a predilection for eumycetoma, while in Mexico actinomycetoma prevails.^18,19^ Hyperendemic countries such as India and Sudan may show geographic variations with respect to epidemiological profiles and pathological spectra of mycetoma.^17,20,21^ Reports of mycetoma outside endemic regions are mostly non-autochthonous, attributed to immigrants originating from endemic areas.^22–26^ However, a recent review by Emery and Denning^27^ suggested worldwide distribution of the disease, with autochthonous cases reported from 102 countries. Nevertheless, knowledge of the true incidence of mycetoma in non-endemic regions remains fragmentary. Several reported cases from Morocco,^28^ Tunisia,^29^ Turkey,^30^ Europe,^31,32^ China^33^ and Egypt^9^ were initially misdiagnosed. This underscores the importance of raising physician and community awareness as well as accurate assessment of disease distribution and burden in non-endemic countries. The recently established Global Mycetoma Working Group aims to address these needs collaboratively and efficiently.^34^

In Africa, mycetoma has been reported in many countries including Sudan, Chad, Niger, Mauritania and Senegal.^27,35^ Sudan is a hyperendemic region for the disease, where >10 000 cases have been published to date.^27^ Surprisingly, in Egypt, which shares its border with Sudan, mycetoma is rarely reported, despite possible mycetoma infection in an ancient Egyptian mummy.^36^ Egypt is the most populous Arab country, with agriculture as one of the main sources of income.^37^ Exposure to environmental aetiologic agents and traumatic implantation and consequent disease burden may be underestimated. As yet, no estimates of the prevalence of mycetoma in Egypt are available. Information on the extent and nature of mycetoma in Egypt may improve clinical practice, change research priorities and allow for estimation of disease burden and appropriate public health policies. Therefore we conducted a scoping literature review to map the evidence of mycetoma in Egypt with emphasis on epidemiology and disease profile.

## Methods

### Research question

This scoping review was conducted according to the guidelines of the Preferred Reporting Items for Systematic Reviews and Meta-Analyses (PRISMA) extension for scoping reviews (PRISMA-ScR; Supplementary file S1).^38^ The main purpose of this scoping review was to explore the extent and nature of previously reported literature on mycetoma infections in Egypt. Additionally, we formulated two questions: What is the amount and type of available literature on mycetoma infections in Egypt? What are the profiles of mycetoma infections in terms of patient and disease characteristics?

### Search strategy

We searched the literature using MEDLINE/PubMed (https://pubmed.ncbi.nlm.nih.gov/) and Google Scholar. The search was extended to include the Embase, Web of Science and Science Direct databases. Additionally, we searched non-peer-reviewed sources or grey literature such as scientific repositories and professional organizational websites (Supplementary file S2). We used the following free-text words and Medical Subject Heading (MeSH) and terms to search the databases: (((((((((((((Mycetoma [MeSH major topic]) OR (Actinomycetoma [MeSH terms])) OR (Eumycetoma [MeSH terms])) OR (Madura Foot [MeSH terms])) OR (Maduromycosis [MeSH terms])) OR (‘actinomycetoma’ [all fields])) OR (‘eumycetoma’ [all fields])) OR (‘madura foot’ [all fields])) OR (‘maduromycosis’ [all fields])) OR (‘mycetoma’ [all fields])) AND (Egypt) (‘mycetoma’[MeSH Major Topic] OR ‘mycetoma’[MeSH Terms] OR ‘mycetoma’[MeSH Terms] OR ‘mycetoma’ [MeSH terms] OR ‘mycetoma’ [MeSH terms] OR ‘actinomycetoma’ [all fields] OR ‘eumycetoma’ [all fields] OR ‘madura foot’ [all fields] OR ‘maduromycosis’ [all fields] OR ‘mycetoma’ [all fields])) OR (dermatomycoses [MeSH major topic])) OR (‘dermatomycoses’ [all fields])) AND (‘Egypt’ [all fields]). The detailed search strategy is shown in Supplementary file S2. The search was conducted on September 2021. We applied the human/species filter to the previous search. We then compared all search results. After screening the identified articles, we searched similar articles and cited by. We did not impose search limitations/filters regarding article type, research design, year of publication or age.

### Retrospective study of patients from a single mycology laboratory

Patients diagnosed with mycetoma at the MT lab (a private laboratory for mycological examination under supervision of Professor Mohamed Taha, Cairo, Egypt) between 2010 and 2013 were reviewed. Patient data within this period of time only was available for review. These patients were referred for mycological examination by dermatologists in Cairo. The referral was based on clinical suspicion of mycetoma. Any case with confirmed mycetoma both clinically and with culture was included in the analysis.

### Data extraction and synthesis of results

For all the papers, the year of study, number of cases, mycetoma type (eu- or actinomycetoma), demographic characteristics of patients (gender, age, nationality), clinical characteristics (site of infection, clinical and radiological features, histopathology), causal agent and diagnostic and management methods (antifungal treatment and/or surgery) were recorded, separately for eu- and actinomycetoma.

## Results

**Figure 1.**
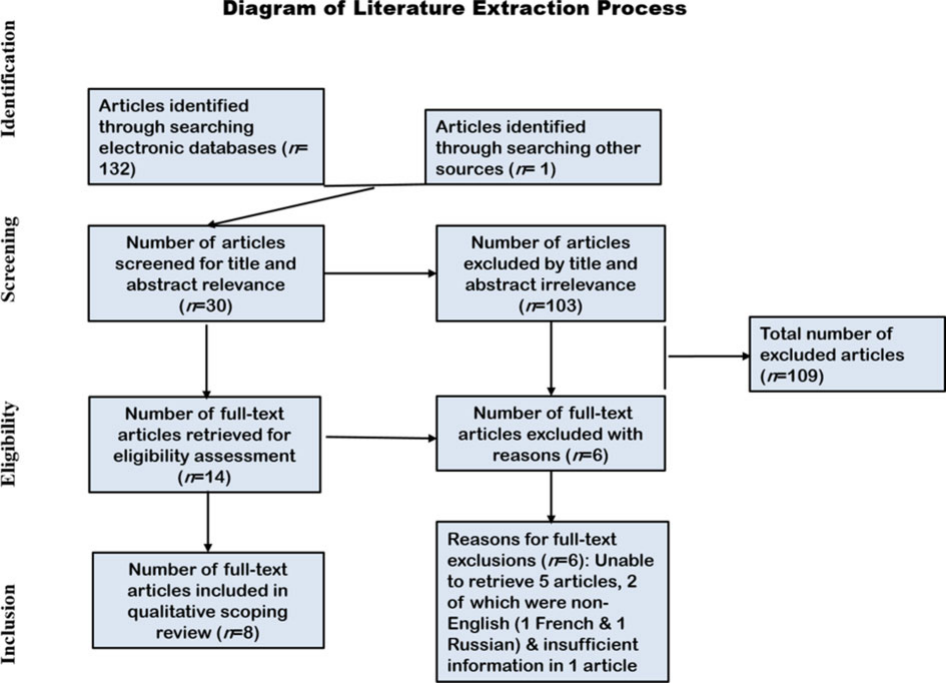
Diagram of the literature extraction process.

The conducted search identified 133 reports, of which 14 were eligible; 6 were excluded due to irretrievability (Figure 1). Eight reports were incorporated in this review. The publication dates of the reports ranged from 1949 to 2015.^9,39–45^ The recorded mycetoma cases involved 59 patients: 44 (75%) eumycetoma patients and 15 (25%) actinomycetoma patients. Four patients (7%) were non-autochthonous/non-native, while 55 (93%) were Egyptians. Three (5.1%) patients were children or adolescents, 52 (88%) were adults and 4 (6.8%) were unreported. Six (10.2%) patients were female, 28 (47.5%) were male and 25 (42.4%) were unreported. Culturing was the most-used diagnostic tool (65.9% in eumycetoma, 40% in actinomycetoma). Therapeutic management was applied with itraconazole or ketoconazole in all 23 eumycetoma cases and surgical management was performed for 2 cases along with antifungal treatment. Antibacterial treatment was the sole management strategy for actinomycetoma.

### Eumycetoma

The 44 eumycetoma patients were reported from Egypt between 1949 and 2015. The demographic and clinical characteristics of reported cases are summarized in Table 1. All 44 patients were residents in Egypt: some were immigrants from Somalia (male), 3 were from Yemen (2 males, 1 female) and 40 were Egyptians (4 females, 20 males, 16 unreported). One patient was 14 y old, while the remaining patients were 18–50 y of age. Infection sites were the foot (n=33), hand (n=5), knee and leg (n=3) and scalp (n=1) and two were without data.

**Table 1.** Spectrum of eumycetoma cases reported in Egypt

Year/reference	Reported patients (n=44)	Sex	Age (years)	Nationality	Site of infection	Clinical and radiological features	Histopathological features	Aetiologic agent	Management
1949/39	1	ND	ND	Egypt	Foot	Multiple cyst-like areas of bone destruction	ND	ND	ND
1963/40	1	ND	ND	Egypt	Foot	ND	ND	ND	ND
1965/41	1	F	35	Egypt	Foot	Granulated ulcers and sinuses with grains 2 mm, destruction and fusion of tarsal and metatarsal bones	Excessive fibrous tissue formation, scanty infiltrates of lymphocytes	*S. boydii*	ND
	1	M	28	Egypt	Foot	Swelling of deformed foot, presence of dark brown granules in seropurulent discharge, osteoporosis of the tarsal bones and obliteration of the intertarsal and tarsometatarsal joints	Masses of granulation tissue and multiple abscesses, granules seen among the abscesses and masses of infiltration	*S. boydii*	ND
1970/42	1	M	27	Egypt	Foot	Multiple swellings with sinuses discharging showing dark brown granules	ND	*S. boydii*	ND
	1	M	32	Egypt	Foot	Multiple swellings with sinuses discharging showing dark brown granules	ND	*M. mycetomatis*	ND
	1	F	18	Egypt	Foot	Multiple swellings with sinuses discharging showing dark brown granules	ND	*M. mycetomatis*	ND
1974/43	2	ND	ND	Egypt	ND	ND	ND	*M. mycetomatis*	ND
2011/44	12	ND	22–81	Egypt	Foot	Large plaques with an atrophic surface studded with multiple sinuses	ND	ND	ND
2010–2013/45	1	M	45	Egypt	Foot	Presence of swellings, small vesicles, abscesses, or boils Fistula with draining of purulent exudates and granules usually predominate	ND	*T. grisea*	Itraconazole after surgery
	8	M	20–50	Egypt	Foot		ND	*M. mycetomatis*	Itraconazole, ketoconazole
	5	3 M, 2 F			Hand				
	1	M		Somalia	Scalp				
	3	2 M, 1 F		Yemen	Knees and legs				
	4	M		Egypt	Foot		ND	No growth	Itraconazole, ketoconazole
2015/9	1	M	14	Egypt	Foot	Painless swelling, multiple sinus tracts developed with initial purulent discharge and an eventual extensive black granular discharge, bony destruction of the calcaneus consistent with chronic osteomyelitis	Appearance of granules surrounded by inflammatory cells and fibrosis	ND	Itraconazole, surgery (resection of the calcaneous)

F, female; M, male; ND, no data.

**Figure 2.**
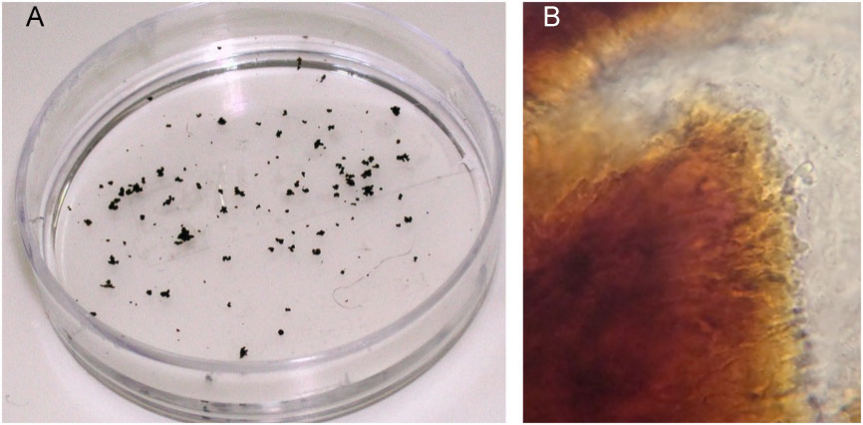
**(A)** Black grains collected from discharging sinuses. **(B)** Direct examination of a crushed grain of eumycetoma (×400).

**Figure 3.**
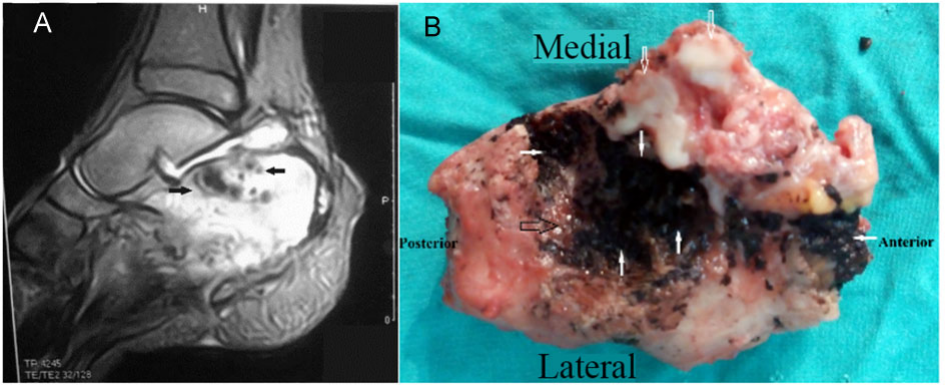
An adolescent boy from rural Egypt with eumycetoma osteomyelitis of calcaneus. **(A)** T2-weighted MRI shows dot-in-circle sign. **(B)** Operative image of superior calcaneus following calcanectomy shows extensive black grains and articular cartilage erosions (hollow white/black arrows). Adopted from El-Sobky et al.^9^

Clinical features were described in 38 cases while radiological features were reported for only three cases. The typical clinical characteristics of mycetoma were described as swellings with multiple sinuses and dark brown grains detected in dark discharge (Figure 2). Radiological findings reported that the tarsal and metatarsal bones were affected,^41^ while bony destruction of the calcaneus was revealed in the child's case (Figure 3).^9^ Histopathological features were described in three cases, appearing as granulation tissue with inflammation. The indicative mycetoma grains appeared in histologic examinations in only two cases.

**Figure 4.**
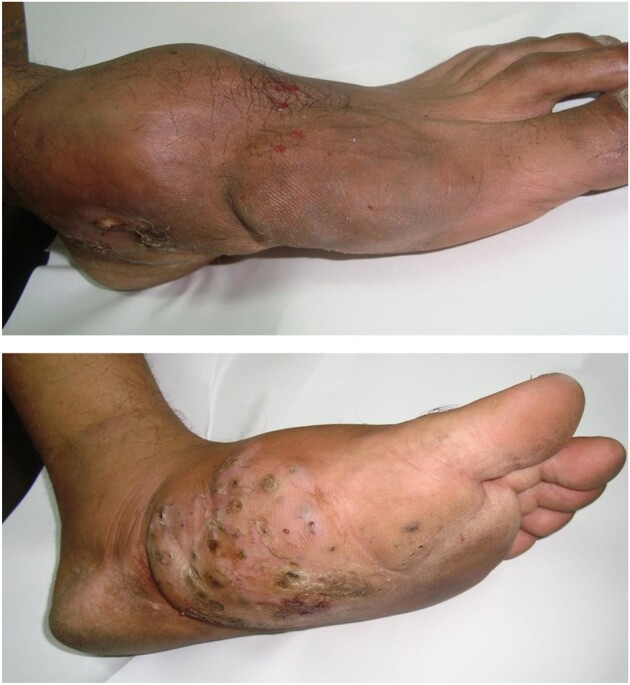
Eumycetoma of the foot caused by *M. mycetomatis*.

The aetiologic agent was cultured in 29 cases, of which the fungus was isolated and identified in 25 cases while 4 cultures showed no growth. *Madurella mycetomatis* was the most common aetiologic agent identified in 21 cases (Figure 4), followed by *Scedosporium boydii* in 3 cases and *Trematosphaeria grisea* in 1 case. Therapeutic management was performed for 23 cases with itraconazole or ketoconazole and surgical management was performed for 2 cases along with antifungal treatment.

### Actinomycetoma

Fifteen actinomycetoma cases were reported from Egypt between 1965 and 2013 (Table 2). All patients were Egyptian, with ages ranging from 15 to 81 y, two patients were female, four were male and nine were unreported. All patients had foot infection.

**Table 2.** Spectrum of actinomycetoma patients reported in Egypt*

Year/reference	Reported patients (n=15)	Sex	Age (years)	Clinical and radiological features	Histopathological features	Aetiologic agents	Management
1965/41	1	F	15	Club-shaped foot, multiple sinuses with underlying soft swellings discharging serosanguinous fluid, yellowish grains measuring 1 mm could be seen in the discharge, osteoporosis of the tarsal bones with obliteration of intertarsal and tarsometatarsal joints	Multiple abscesses surrounded by excessive granulation tissue that was infiltrated with histiocytes, fibroblasts, neutrophils and plasma cells	*Nocardia* sp.	ND
2011/44	9	ND	22–81	ND	ND	ND	ND
2010–2013/45	4	3 M, 1 F	20–50	Presence of swellings, small vesicles, abscesses or boils, fistula with draining of purulent exudates and granules usually predominate	ND	*A. madurae*	Trimethoprime-sulphamethoxazole, amoxicillin clavulanic acid and amikacin
	1	M	20–50		ND	*N. asteroides*	Ciprofloxacin and erythromycin

F, female; M, male; ND, no data.

* All patients were autochthonous or Egyptians and site of infection was the foot in all patients.

Clinical features were described in six cases; one report^41^ added radiological characteristics, showing that tarsal and metatarsal joints were affected. The clinical appearance of actinomycetoma was described as a club-shaped foot and multiple sinuses with underlying soft swellings discharging serosanguinous fluid. Yellowish grains measuring 1 mm could be seen in the discharge. Histopathological features were described by el Mofty et al.,^41^ appearing as multiple abscesses surrounded by excessive granulation tissue that was infiltrated with histiocytes, fibroblasts, neutrophils and plasma cells.

**Figure 5.**
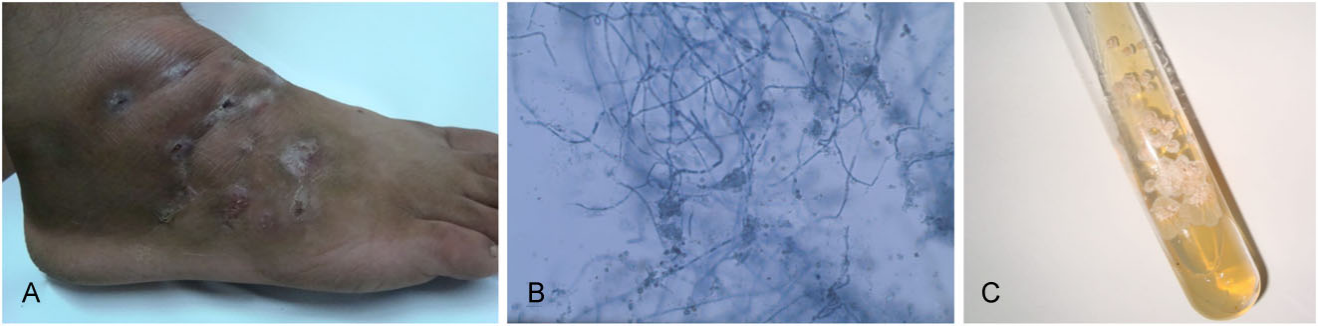
**(A)** Actinomycetoma of the foot caused by *N. asteroides*. **(B)** Lactophenol cotton blue preparation and **(C)** culture of *N. asteroides* from white grain actinomycetoma.

Isolation of the aetiologic agents was carried out in six patients and the obtained actinomycetes isolates identified as *Actinomadura madurae* in four patients and both *Nocardia* sp. and *Nocardia asteroides* in one patient each (Figure 5).

Therapeutic management was performed for five patients, where trimethoprime-sulphamethoxazole, amoxicillin, clavulanic acid and amikacin were used in the four cases caused by *A. madurae* and ciprofloxacin and erythromycin were used in the case caused by *N. asteroides*.

## Discussion

### Summary of evidence

**Figure 6.**
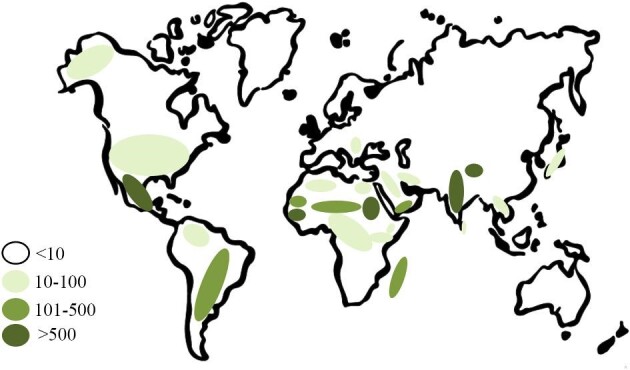
Mycetoma world map revisited. Note the inclusion of Egypt as a low-endemic country. Figure redrawn from Emery and Denning.^27^

This scoping review found definite evidence for autochthonous mycetoma infections in Egypt. Additionally, non-autochthonous mycetoma patients were recognized as well. Both actinomycetoma and eumycetoma were reported, with a special predilection toward the latter. There is relatively scant literature to provide a precise estimate of its national or regional prevalence across Egypt. Nevertheless, this review spotlighted the disease and patient demographics that otherwise have not been reported previously.^27,35^ Further, this review shows that there are grounds for believing that autochthonous mycetoma infections in Egypt are not uncommon and that disease burden is higher than previously thought.^27^ In this respect, we reported a total number of mycetoma patients (59 patients) that is higher than previously reported by Emery and Denning,^27^ who investigated the global distribution of actinomycetoma and eumycetoma. This may be attributed to the capture of non-peer-reviewed literature with ample cases,^45^ among other reasons. Accordingly, we have updated the map published by Emery and Denning^27^ and proposed new categories based on the number of published cases in each country. Countries with >500 cases are probably highly endemic regions, while moderately endemic areas involve countries with 101–500 cases. Countries with 51–100 cases are probably low endemic, while areas with <50 cases represent non-endemic regions (Figure 6). Thus Egypt might still be considered as endemic for mycetoma, and researching the true prevalence of the disease is justifiable and may be clinically worthwhile.

**Figure 7.**
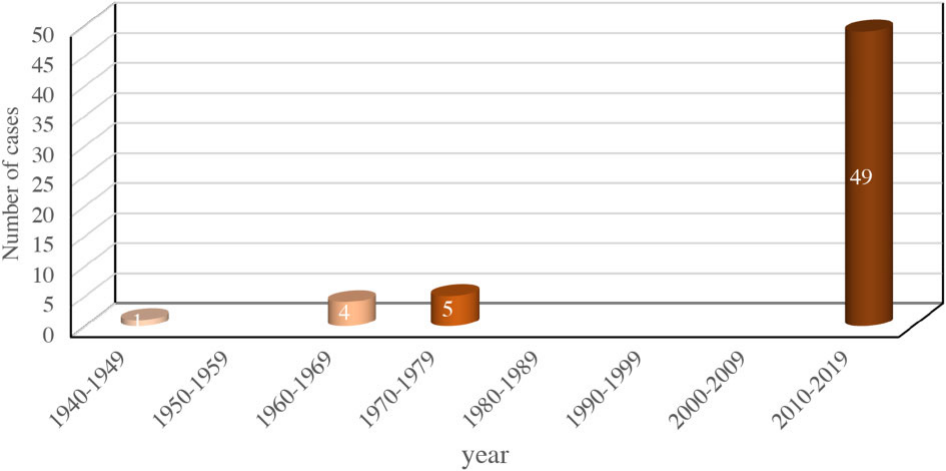
The number of published mycetoma cases in Egypt for the period of 1940–2021.

The assumption that the prevalence of mycetoma in Egypt is underestimated has circumstantial evidence in its favour. For example, physician unawareness can be a pivotal contributing factor.^13^ Further, patients with suspected mycetoma infection require unhindered referral to a specialized centre equipped with all necessary tools and multidiscipline expertise so as to confirm the diagnosis and identify the causal pathogen.^15^ In this regard, physician unawareness and the lack of an accessible specialized mycetoma centre in Egypt can potentially contribute to misdiagnosis, which in turn can give false estimates of nationwide mycetoma prevalence and demographics. Remarkably, only 10 cases of the disease were reported during the period 1940–2009 (Figure 7). The numbers after 2010 were considerably higher, but we could not retrieve any case after 2015. The substandard medical records in general and the lack of electronic records, at least in rural areas, can fail to preserve data for suspected or documented mycetoma patients. Further, this does not allow for the performance of quality retrospective review research of the medical records.

In this review, eumycetoma was the predominant type in Egypt, representing 75% of reported patients, while actinomycetoma constituted 25%. This conforms to the demographics of the disease in the neighbouring country Sudan.^20^ Likewise, the most common site of infection in our study was the foot, which is consistent with the findings of studies arising from countries on the African continent.^7,16,20,46^ Our results revealed that *M. mycetomatis* was the most common aetiologic agent of the disease in Egypt. This fungus also presents as the most prevalent agent in Sudan.^20^ The lower number of cases in Egypt compared with Sudan might be due to the presence of certain genetic predispositions toward *M. mycetomatis* infection in the Sudanese population or better construction of villages in Egypt and the use of footwear. We also found *A. madurae* as a common agent of actinomycetoma, identified in 6 of 15 reported actinomycetoma patients. These results did not contradict those confirmed in previous studies from Africa.^15,16,46^

### Study implications

The inclusion of Egypt in global mycetoma campaigns may bring public health and clinical benefits. First, it will increase physician awareness in both primary and specialist domains. Second, it will enhance further research collaboration exploring both public health and clinical aspects of mycetoma.^47^ Furthermore, the Nile Valley, namely Sudan and Egypt, represents a broad geopolitical and cultural continuum with liberal cross-border human travel. This should raise concerns about non-autochthonous mycetoma patients imported from high-endemicity zones in Sudan to Egypt and necessitates mycetoma health awareness campaigns geared towards Egyptian physicians across multiple specialties.

The differential diagnosis of soft tissue and bone infections of the foot and leg is of the greatest importance. This is because they are the most common site of mycetoma infections. Unlike bacterial osteomyelitis and septic arthritis, mycetoma infections are generally related to certain occupations, such as farming and herd raising, that may entail working barefooted.^48,49^ This calls for wide proactive measures aimed at raising the awareness of vulnerable populations regarding the use of protective clothes and footwear, among others. This is of particular importance not only for mycetoma, but also for other mycotic diseases, because the Middle East and North Africa in general, and Egypt specifically, are postulated to have a considerable burden of serious fungal infections.^50,51^

### Study limitations

We acknowledge several inherent study limitations. Some studies had one or more missing pieces of information, e.g. histopathology, radiologic features, treatment details (whether medical, surgical or combined) and autochthonous versus non-autochthonous patients. Some studies did not verify the diagnosis with a microbiological or fungal culture. Six articles were primarily eligible for inclusion yet were irretrievable. The previous factors may have collectively influenced the evidence upon which the conclusions were dependent. Of note, some studies were dated, and it is likely that recent laboratory tools and MRI were not available for the diagnostic workup. It should be noted that molecular identification, which may be the only tool to precisely identify the causative agent, was not performed in any of the reviewed studies. Nevertheless, most of the reported patients were evaluated by mycologists with credible clinical expertise.

## Conclusions

The definite presence of mycetoma infections in Egypt has been confirmed and its clinical and demographic features described within the confines of the relatively small numbers reported. The true prevalence and national demographics of mycetoma was impossibly difficult to estimate. However, there is mounting presumptive evidence that the prevalence of mycetoma in Egypt is considerably underreported and underestimated. This calls forth a multitude of recommendations. First, relevant academic departments should take administrative steps to prioritize clinical research on topics related to mycetoma in Egypt. Second, mycology units should adopt a multidisciplinary approach to fungal infections in general and mycetoma specifically, incorporating mycologists, dermatologists, pathologists and surgeons, among others. Third, individual researchers and institutions should establish links with the relevant international experts and organizations such as the WHO and its collaborating centres aimed at holding health awareness campaigns, allocating funds and initiating research collaborations related to mycetoma in Egypt.

## Supplementary Material

trac085_Supplemental_Files

## Data Availability

The authors confirm that the data supporting the findings of this study are available within the article and its supplementary materials.
