# Supplementary material for: A scoping review of mycetoma profile in Egypt: revisiting the global endemicity map
Source: Trans R Soc Trop Med Hyg. 2022 Sep 9;117(1):1–11. doi: 10.1093/trstmh/trac085 (PMC9808524; doi:10.1093/trstmh/trac085)
Supplement: trac085_Supplemental_Files [file trac085_supplemental_files.zip › Supplementary File S2 Revision.docx]

**A Scoping Review of Mycetoma Profile in Egypt: Revisiting the Global Endemicity Map**

**Sarah A. Ahmed^1,2*^, Tamer A. El-Sobky^3*^, Sybren de Hoog^1,2,4,5^, Sherif M. Zaki^6^, Mohamed Taha^7^**

*^1^Center of Expertise in Mycology Radboudumc / Canisius Wilhelmina Hospital, Nijmegen, The Netherlands*

*^2^Foundation Atlas of Clinical Fungi, Hilversum, The Netherlands*

*^3^Division of Pediatric Orthopaedics, Department of Orthopedic Surgery, Faculty of Medicine, Ain Shams University, Cairo, Egypt*

*^4^Department of Basic Pathology, Federal University of Paraná State, Curitiba, Brazil*

*^5^Department of Dermatology, Peking University First Hospital, Research Center for Medical Mycology, Peking University, Beijing, China*

*^6^Mycology Unit, Department of Microbiology, Faculty of Science, Ain Shams University, Cairo, Egypt*

*^7^Department of Microbiology, Zagazig University, Cairo, Egypt*

**Search Strategy** <https://pubmed.ncbi.nlm.nih.gov/>

- Search results from the National Center for Biotechnology Information (NCBI) at the U.S. National Library of Medicine (NLM) conducted On: Sep 26, 2021

Search: **(((((((((((((Mycetoma[MeSH Major Topic]) OR (Actinomycetoma[MeSH Terms])) OR (Eumycetoma[MeSH Terms])) OR (Madura Foot[MeSH Terms])) OR (Maduromycosis[MeSH Terms])) OR ("actinomycetoma"[All Fields])) OR ("eumycetoma"[All Fields])) OR ("madura foot"[All Fields])) OR ("maduromycosis"[All Fields])) OR ("mycetoma"[All Fields])) AND (Egypt) ("mycetoma"[MeSH Major Topic] OR "mycetoma"[MeSH Terms] OR "mycetoma"[MeSH Terms] OR "mycetoma"[MeSH Terms] OR "mycetoma"[MeSH Terms] OR "actinomycetoma"[All Fields] OR "eumycetoma"[All Fields] OR "madura foot"[All Fields] OR "maduromycosis"[All Fields] OR "mycetoma"[All Fields]) ) OR (Dermatomycoses[MeSH Major Topic])) OR ("dermatomycoses"[All Fields])) AND ("egypt"[All Fields])**

(((("mycetoma"[MeSH Major Topic] OR "mycetoma"[MeSH Terms] OR "mycetoma"[MeSH Terms] OR "mycetoma"[MeSH Terms] OR "mycetoma"[MeSH Terms] OR "actinomycetoma"[All Fields] OR "eumycetoma"[All Fields] OR "madura foot"[All Fields] OR "maduromycosis"[All Fields] OR "mycetoma"[All Fields]) AND ("egypt"[MeSH Terms] OR "egypt"[All Fields] OR "egypt s"[All Fields])) AND ("mycetoma"[MeSH Major Topic] OR "mycetoma"[MeSH Terms] OR "mycetoma"[MeSH Terms] OR "mycetoma"[MeSH Terms] OR "mycetoma"[MeSH Terms] OR "actinomycetoma"[All Fields] OR "eumycetoma"[All Fields] OR "madura foot"[All Fields] OR "maduromycosis"[All Fields] OR "mycetoma"[All Fields])) OR "dermatomycoses"[MeSH Major Topic] OR "dermatomycoses"[All Fields]) AND "egypt"[All Fields]

**Translations**

**Mycetoma[MeSH Major Topic]:** "mycetoma"[MeSH Major Topic]

**Actinomycetoma[MeSH Terms]:** "mycetoma"[MeSH Terms]

**Eumycetoma[MeSH Terms]:** "mycetoma"[MeSH Terms]

**Madura Foot[MeSH Terms]:** "mycetoma"[MeSH Terms]

**Maduromycosis[MeSH Terms]:** "mycetoma"[MeSH Terms]

**Egypt:** "egypt"[MeSH Terms] OR "egypt"[All Fields] OR "egypt's"[All Fields]

**Dermatomycoses[MeSH Major Topic]:** "dermatomycoses"[MeSH Major Topic]

Items 1-131 of 131 ([Display the 131 citations in PubMed](https://pubmed.ncbi.nlm.nih.gov/?term=26692117,31633447,29239242,32304603,20718613,31502751,31728627,28327256,14314419,22575159,25330098,30946748,27671284,33148275,31157575,26261140,26483983,32667111,32182387,31025207,30775844,28188051,31493312,32975877,20849434,25481439,32266837,31511056,30081698,26707860,33141453,19767633,30554365,24129248,26447337,26413063,18972221,21506964,25953414,29314351,32162164,29768066,23760076,12916729,27654953,16681816,27264960,31697010,33404930,28522423,27242275,26296782,28339548,21725708,8737885,27666873,4249711,25669435,8935633,16916049,25234393,27337493,8919935,24336943,22151832,7999601,4474964,18579714,27549079,18798033,26135513,5425322,29952296,7591430,7960360,8546171,26576042,16230557,1265571,5464089,4497480,12000511,28446137,26948078,27392537,7960355,12197355,12206316,4564495,913867,5761179,5110393,5796000,7928033,14127037,14162103,7263125,16886418,14162117,7984217,8150398,14314418,18143687,9466206,5595806,5575840,4771479,1176246,6872104,13321236,997879,13993651,13070299,14080707,14162097,14162112,14461148,4636066,13752862,5075545,14162090,5548033,5102750,14928252,5595373,6948679,14176377,5650626,13819849,13184069,765093))

| 1. | [Dermatophytosis in northern Africa.](https://pubmed.ncbi.nlm.nih.gov/26692117/)  Nweze EI, Eke I.  Mycoses. 2016 Mar;59(3):137-44. doi: 10.1111/myc.12447. Epub 2015 Dec 22.  PMID: 26692117 Review. |
| --- | --- |
| 2. | [The potential role of interleukin-37 in infectious diseases.](https://pubmed.ncbi.nlm.nih.gov/31633447/)  Allam G, Gaber AM, Othman SI, Abdel-Moneim A.  Int Rev Immunol. 2020;39(1):3-10. doi: 10.1080/08830185.2019.1677644. Epub 2019 Oct 21.  PMID: 31633447 Review. |
| 3. | [Fluconazole-loaded solid lipid nanoparticles topical gel for treatment of pityriasis versicolor: formulation and clinical study.](https://pubmed.ncbi.nlm.nih.gov/29239242/)  El-Housiny S, Shams Eldeen MA, El-Attar YA, Salem HA, Attia D, Bendas ER, El-Nabarawi MA.  Drug Deliv. 2018 Nov;25(1):78-90. doi: 10.1080/10717544.2017.1413444.  PMID: 29239242 **Free PMC article.** Clinical Trial. |
| 4. | [In vitro antifungal susceptibility testing of fungi in patients with onychomycosis.](https://pubmed.ncbi.nlm.nih.gov/32304603/)  Abu El-Hamd M, Abd Elhameed MI, Shalaby MFM, Saleh R.  Dermatol Ther. 2020 May;33(3):e13429. doi: 10.1111/dth.13429. Epub 2020 May 8.  PMID: 32304603 |
| 5. | [Invasive aspergillosis in developing countries.](https://pubmed.ncbi.nlm.nih.gov/20718613/)  Chakrabarti A, Chatterjee SS, Das A, Shivaprakash MR.  Med Mycol. 2011 Apr;49 Suppl 1:S35-47. doi: 10.3109/13693786.2010.505206. Epub 2010 Aug 18.  PMID: 20718613 Review. |
| 6. | [Onychomycosis: Correlation between the dermoscopic patterns and fungal culture.](https://pubmed.ncbi.nlm.nih.gov/31502751/)  Abdallah NA, Said M, Mahmoud MT, Omar MA.  J Cosmet Dermatol. 2020 May;19(5):1196-1204. doi: 10.1111/jocd.13144. Epub 2019 Sep 10.  PMID: 31502751 |
| 7. | [Diagnosis of onychomycosis clinically by nail dermoscopy versus microbiological diagnosis.](https://pubmed.ncbi.nlm.nih.gov/31728627/)  Nada EEA, El Taieb MA, El-Feky MA, Ibrahim HM, Hegazy EM, Mohamed AE, El-Amir MI.  Arch Dermatol Res. 2020 Apr;312(3):207-212. doi: 10.1007/s00403-019-02008-6. Epub 2019 Nov 14.  PMID: 31728627 |
| 8. | [Origin and distribution of Sporothrix globosa causing sapronoses in Asia.](https://pubmed.ncbi.nlm.nih.gov/28327256/)  Moussa TAA, Kadasa NMS, Al Zahrani HS, Ahmed SA, Feng P, Gerrits van den Ende AHG, Zhang Y, Kano R, Li F, Li S, Song Y, Dong B, Rossato L, Dolatabadi S, Hoog S.  J Med Microbiol. 2017 May;66(5):560-569. doi: 10.1099/jmm.0.000451. Epub 2017 May 22.  PMID: 28327256 Review. |
| 9. | [MADURA FOOT" IN EGYPT.](https://pubmed.ncbi.nlm.nih.gov/14314419/)  EL-MOFTY AM, ISKANDER IO, NADA M, ZAKI SM.  Br J Dermatol. 1965 Jul;77:365-72. doi: 10.1111/j.1365-2133.1965.tb14662.x.  PMID: 14314419 No abstract available. |
| 10. | [Hair loss in pityriasis versicolor lesions: a descriptive clinicopathological study.](https://pubmed.ncbi.nlm.nih.gov/22575159/)  Mostafa WZ, Assaf MI, Ameen IA, El Safoury OS, Al Sulh SA.  J Am Acad Dermatol. 2013 Jul;69(1):e19-23. doi: 10.1016/j.jaad.2012.03.004. Epub 2012 May 8.  PMID: 22575159 |
| 11. | [Mapping the potential risk of mycetoma infection in Sudan and South Sudan using ecological niche modeling.](https://pubmed.ncbi.nlm.nih.gov/25330098/)  Samy AM, van de Sande WW, Fahal AH, Peterson AT.  PLoS Negl Trop Dis. 2014 Oct 16;8(10):e3250. doi: 10.1371/journal.pntd.0003250. eCollection 2014 Oct.  PMID: 25330098 **Free PMC article.** |
| 12. | [The Role of Interleukin-1 cytokine family (IL-1β, IL-37) and interleukin-12 cytokine family (IL-12, IL-35) in eumycetoma infection pathogenesis.](https://pubmed.ncbi.nlm.nih.gov/30946748/)  Abushouk A, Nasr A, Masuadi E, Allam G, Siddig EE, Fahal AH.  PLoS Negl Trop Dis. 2019 Apr 4;13(4):e0007098. doi: 10.1371/journal.pntd.0007098. eCollection 2019 Apr.  PMID: 30946748 **Free PMC article.** |
| 13. | [Pyocyanin as anti-tyrosinase and anti tinea corporis: A novel treatment study.](https://pubmed.ncbi.nlm.nih.gov/27671284/)  El-Zawawy NA, Ali SS.  Microb Pathog. 2016 Nov;100:213-220. doi: 10.1016/j.micpath.2016.09.013. Epub 2016 Sep 23.  PMID: 27671284 Review. |
| 14. | [Ringworm in calves: risk factors, improved molecular diagnosis, and therapeutic efficacy of an Aloe vera gel extract.](https://pubmed.ncbi.nlm.nih.gov/33148275/)  Tartor YH, El-Neshwy WM, Merwad AMA, Abo El-Maati MF, Mohamed RE, Dahshan HM, Mahmoud HI.  BMC Vet Res. 2020 Nov 4;16(1):421. doi: 10.1186/s12917-020-02616-9.  PMID: 33148275 **Free PMC article.** |
| 15. | [Combined long-pulsed Nd-Yag laser and itraconazole versus itraconazole alone in the treatment of onychomycosis nails.](https://pubmed.ncbi.nlm.nih.gov/31157575/)  Hamed Khater M, Khattab FM.  J Dermatolog Treat. 2020 Jun;31(4):406-409. doi: 10.1080/09546634.2019.1623861. Epub 2019 Jun 14.  PMID: 31157575 Clinical Trial. |
| 16. | [Recent advances in topical formulation carriers of antifungal agents.](https://pubmed.ncbi.nlm.nih.gov/26261140/)  Bseiso EA, Nasr M, Sammour O, Abd El Gawad NA.  Indian J Dermatol Venereol Leprol. 2015 Sep-Oct;81(5):457-63. doi: 10.4103/0378-6323.162328.  PMID: 26261140 Review. |
| 17. | [Eumycetoma Osteomyelitis of the Calcaneus in a Child: A Radiologic-Pathologic Correlation following Total Calcanectomy.](https://pubmed.ncbi.nlm.nih.gov/26483983/)  El-Sobky TA, Haleem JF, Samir S.  Case Rep Pathol. 2015;2015:129020. doi: 10.1155/2015/129020. Epub 2015 Sep 21.  PMID: 26483983 **Free PMC article.** |
| 18. | [Successful treatment of resistant onychomycosis with voriconazole in a liver transplant patient.](https://pubmed.ncbi.nlm.nih.gov/32667111/)  Nofal A, Fawzy MM, El-Hawary EE.  Dermatol Ther. 2020 Nov;33(6):e14014. doi: 10.1111/dth.14014. Epub 2020 Aug 12.  PMID: 32667111 |
| 19. | [Adapalene gel 0.1% vs ketoconazole cream 2% and their combination in treatment of pityriasis versicolor: A randomized clinical study.](https://pubmed.ncbi.nlm.nih.gov/32182387/)  Bakr E, Abdo H, Abd-Elaziz H, Abd-Elrazek H, Amer M.  Dermatol Ther. 2020 May;33(3):e13319. doi: 10.1111/dth.13319. Epub 2020 Mar 30.  PMID: 32182387 Clinical Trial. |
| 20. | [Fractional carbon dioxide laser and topical tioconazole in the treatment of fingernail onychomycosis.](https://pubmed.ncbi.nlm.nih.gov/31025207/)  El-Tatawy RA, Aliweh HA, Hegab DS, Talaat RAZ, Shams Eldeen MA.  Lasers Med Sci. 2019 Dec;34(9):1873-1880. doi: 10.1007/s10103-019-02789-2. Epub 2019 Apr 25.  PMID: 31025207 |
| 21. | [Trichophyton mentagrophytes - a new genotype of zoophilic dermatophyte causes sexually transmitted infections.](https://pubmed.ncbi.nlm.nih.gov/30775844/)  Kupsch C, Czaika VA, Deutsch C, Gräser Y.  J Dtsch Dermatol Ges. 2019 May;17(5):493-501. doi: 10.1111/ddg.13776. Epub 2019 Feb 18.  PMID: 30775844 |
| 22. | [Incidence and biodiversity of yeasts, dermatophytes and non-dermatophytes in superficial skin infections in Assiut, Egypt.](https://pubmed.ncbi.nlm.nih.gov/28188051/)  Moubasher AH, Abdel-Sater MA, Soliman Z.  J Mycol Med. 2017 Jun;27(2):166-179. doi: 10.1016/j.mycmed.2017.01.005. Epub 2017 Feb 7.  PMID: 28188051 |
| 23. | [Towards a rapid identification and a novel proteomic analysis for dermatophytes from human and animal dermatophytosis.](https://pubmed.ncbi.nlm.nih.gov/31493312/)  Tartor YH, Abo Hashem ME, Enany S.  Mycoses. 2019 Dec;62(12):1116-1126. doi: 10.1111/myc.12998. Epub 2019 Oct 16.  PMID: 31493312 |
| 24. | [Comparative study between the effect of topical tazarotene 0.1 gel alone vs its combination with tioconazole nail paint in treatment of onychomycosis.](https://pubmed.ncbi.nlm.nih.gov/32975877/)  El-Salam SSA, Omar GA, Mahmoud MT, Said M.  Dermatol Ther. 2020 Nov;33(6):e14333. doi: 10.1111/dth.14333. Epub 2020 Oct 9.  PMID: 32975877 |
| 25. | [Skin infections in Egyptian renal transplant recipients.](https://pubmed.ncbi.nlm.nih.gov/20849434/)  Bakr NI, El-Sawy E, Hamdy AF, Bakr MA.  Transpl Infect Dis. 2011 Apr;13(2):131-5. doi: 10.1111/j.1399-3062.2010.00568.x. Epub 2010 Sep 16.  PMID: 20849434 |
| 26. | [Development of topical therapeutics for management of onychomycosis and other nail disorders: a pharmaceutical perspective.](https://pubmed.ncbi.nlm.nih.gov/25481439/)  Elsayed MM.  J Control Release. 2015 Feb 10;199:132-44. doi: 10.1016/j.jconrel.2014.11.017. Epub 2014 Dec 4.  PMID: 25481439 Review. |
| 27. | [Oxiconazole nitrate solid lipid nanoparticles: formulation, in-vitro characterization and clinical assessment of an analogous loaded carbopol gel.](https://pubmed.ncbi.nlm.nih.gov/32266837/)  Mahmoud RA, Hussein AK, Nasef GA, Mansour HF.  Drug Dev Ind Pharm. 2020 May;46(5):706-716. doi: 10.1080/03639045.2020.1752707. Epub 2020 Apr 15.  PMID: 32266837 Clinical Trial. |
| 28. | [Identifying asymptomatic Leishmania infections in non-endemic villages in Gedaref state, Sudan.](https://pubmed.ncbi.nlm.nih.gov/31511056/)  Mohamed NS, Osman HA, Muneer MS, Samy AM, Ahmed A, Mohammed AO, Siddig EE, Abdel Hamid MM, Ali MS, Omer RA, Elaagip AH.  BMC Res Notes. 2019 Sep 11;12(1):566. doi: 10.1186/s13104-019-4608-2.  PMID: 31511056 **Free PMC article.** |
| 29. | [Fractional carbon dioxide laser assisted delivery of topical tazarotene versus topical tioconazole in the treatment of onychomycosis.](https://pubmed.ncbi.nlm.nih.gov/30081698/)  Abd El-Aal EB, Abdo HM, Ibrahim SM, Eldestawy MT.  J Dermatolog Treat. 2019 May;30(3):277-282. doi: 10.1080/09546634.2018.1509046. Epub 2018 Sep 28.  PMID: 30081698 Clinical Trial. |
| 30. | [Control of tinea capitis.](https://pubmed.ncbi.nlm.nih.gov/26707860/)  Gohar A.  Br J Dermatol. 2016 May;174(5):1158. doi: 10.1111/bjd.14372. Epub 2016 Mar 14.  PMID: 26707860 No abstract available. |
| 31. | [Trichoscopic signs of tinea capitis: a guide for selection of appropriate antifungal.](https://pubmed.ncbi.nlm.nih.gov/33141453/)  Genedy RM, Sorour OA, Elokazy MAW.  Int J Dermatol. 2021 Apr;60(4):471-481. doi: 10.1111/ijd.15289. Epub 2020 Nov 3.  PMID: 33141453 |
| 32. | [Nail changes in kidney transplant recipients.](https://pubmed.ncbi.nlm.nih.gov/19767633/)  Abdelaziz AM, Mahmoud KM, Elsawy EM, Bakr MA.  Nephrol Dial Transplant. 2010 Jan;25(1):274-7. doi: 10.1093/ndt/gfp486. Epub 2009 Sep 19.  PMID: 19767633 |
| 33. | [Comparative effectiveness of Sumaq and Neem extract cream, Eniloconazole and glycerine iodine on dermatophytosis in Arabian horses: a randomized clinical trial.](https://pubmed.ncbi.nlm.nih.gov/30554365/)  Sayed-Ahmed MZ, Ahdy AM, Younis EE, El-Khodery SA, Baraka HN.  Trop Anim Health Prod. 2019 May;51(4):905-910. doi: 10.1007/s11250-018-1773-6. Epub 2018 Dec 15.  PMID: 30554365 Clinical Trial. |
| 34. | [Efficacy of some synthesized thiazoles against dermatophytes.](https://pubmed.ncbi.nlm.nih.gov/24129248/)  Ouf SA, Taleb AM, Tharwat NA, Geweely NS.  J Mycol Med. 2013 Dec;23(4):230-6. doi: 10.1016/j.mycmed.2013.07.056. Epub 2013 Oct 12.  PMID: 24129248 |
| 35. | [Novel nail penetration enhancer containing vesicles "nPEVs" for treatment of onychomycosis.](https://pubmed.ncbi.nlm.nih.gov/26447337/)  Bseiso EA, Nasr M, Sammour OA, Abd El Gawad NA.  Drug Deliv. 2016 Oct;23(8):2813-2819. doi: 10.3109/10717544.2015.1099059. Epub 2015 Oct 8.  PMID: 26447337 |
| 36. | [Dermatophytes and other associated fungi in patients attending to some hospitals in Egypt.](https://pubmed.ncbi.nlm.nih.gov/26413063/)  Abd Elmegeed AS, Ouf SA, Moussa TA, Eltahlawi SM.  Braz J Microbiol. 2015 Jul 1;46(3):799-805. doi: 10.1590/S1517-838246320140615. eCollection 2015 Jul-Sep.  PMID: 26413063 **Free PMC article.** |
| 37. | [Dermatophyte infections in Cairo, Egypt.](https://pubmed.ncbi.nlm.nih.gov/18972221/)  Zaki SM, Ibrahim N, Aoyama K, Shetaia YM, Abdel-Ghany K, Mikami Y.  Mycopathologia. 2009 Mar;167(3):133-7. doi: 10.1007/s11046-008-9165-5. Epub 2008 Oct 30.  PMID: 18972221 |
| 38. | [Cowden's syndrome: a clinical, immunological, and histopathological study.](https://pubmed.ncbi.nlm.nih.gov/21506964/)  Amer M, Mostafa FF, Attwa EM, Ibrahim S.  Int J Dermatol. 2011 May;50(5):516-21. doi: 10.1111/j.1365-4632.2010.04669.x.  PMID: 21506964 |
| 39. | [Anti-dermatophyte efficacy and environmental safety of some essential oils commercial and in vitro extracted pure and combined against four keratinophilic pathogenic fungi.](https://pubmed.ncbi.nlm.nih.gov/25953414/)  Ibrahim SY, Abd El-Salam MM.  Environ Health Prev Med. 2015 Jul;20(4):279-86. doi: 10.1007/s12199-015-0462-6. Epub 2015 May 8.  PMID: 25953414 **Free PMC article.** |
| 40. | [Epidemiology of dermatophyte infections among school children in Menoufia Governorate, Egypt.](https://pubmed.ncbi.nlm.nih.gov/29314351/)  Farag AGA, Hammam MA, Ibrahem RA, Mahfouz RZ, Elnaidany NF, Qutubuddin M, Tolba RRE.  Mycoses. 2018 May;61(5):321-325. doi: 10.1111/myc.12743. Epub 2018 Jan 23.  PMID: 29314351 |
| 41. | [Comparative study between topically applied irradiated human amniotic membrane in combination with tea tree oil versus topical tioconazole in pityraisis versicolor treatment.](https://pubmed.ncbi.nlm.nih.gov/32162164/)  Nashwa RK, Ahmed EB, Nemr WA.  Cell Tissue Bank. 2020 Jun;21(2):313-320. doi: 10.1007/s10561-020-09824-5. Epub 2020 Mar 12.  PMID: 32162164 |
| 42. | [Evaluation of long pulsed Nd-YAG laser in the treatment of onychomycosis.](https://pubmed.ncbi.nlm.nih.gov/29768066/)  Ibrahim SA, Albalat W, Ebrahim HM.  J Cosmet Laser Ther. 2019;21(2):76-81. doi: 10.1080/14764172.2018.1469765. Epub 2018 May 16.  PMID: 29768066 No abstract available. |
| 43. | [Public health significance of dermatophytes in Ismailia and Port Said Provinces, Egypt.](https://pubmed.ncbi.nlm.nih.gov/23760076/)  Aboueisha AM, El-Mahallawy H.  Med Mycol J. 2013;54(2):123-9. doi: 10.3314/mmj.54.123.  PMID: 23760076 |
| 44. | [Study of solar photosensitization processes on dermatophytic fungi.](https://pubmed.ncbi.nlm.nih.gov/12916729/)  Ouf SA, Abdel-Kader MH, Shokeir HA, El-Adly AA.  Acta Microbiol Pol. 2003;52(1):65-79.  PMID: 12916729 |
| 45. | [Trichoscopy as a useful method to differentiate tinea capitis from alopecia areata in children at Zagazig University Hospitals.](https://pubmed.ncbi.nlm.nih.gov/27654953/)  Amer M, Helmy A, Amer A.  Int J Dermatol. 2017 Jan;56(1):116-120. doi: 10.1111/ijd.13217. Epub 2016 Sep 22.  PMID: 27654953 |
| 46. | [Efficacy of topical griseofulvin in treatment of tinea corporis.](https://pubmed.ncbi.nlm.nih.gov/16681816/)  Kassem MA, Esmat S, Bendas ER, El-Komy MH.  Mycoses. 2006 May;49(3):232-5. doi: 10.1111/j.1439-0507.2006.01221.x.  PMID: 16681816 Clinical Trial. |
| 47. | [When to suspect tinea; a histopathologic study of 103 cases of PAS-positive tinea.](https://pubmed.ncbi.nlm.nih.gov/27264960/)  Elbendary A, Valdebran M, Gad A, Elston DM.  J Cutan Pathol. 2016 Oct;43(10):852-7. doi: 10.1111/cup.12757. Epub 2016 Jul 12.  PMID: 27264960 |
| 48. | [Fractional CO_2_ laser plus topical antifungal versus fractional CO_2_ laser versus topical antifungal in the treatment of onychomycosis.](https://pubmed.ncbi.nlm.nih.gov/31697010/)  Zaki AM, Abdo HM, Ebadah MA, Ibrahim SM.  Dermatol Ther. 2020 Jan;33(1):e13155. doi: 10.1111/dth.13155. Epub 2019 Nov 14.  PMID: 31697010 Clinical Trial. |
| 49. | [Effect of Different Nail Penetration Enhancers in Solid Lipid Nanoparticles Containing Terbinafine Hydrochloride for Treatment of Onychomycosis.](https://pubmed.ncbi.nlm.nih.gov/33404930/)  Abobakr FE, Fayez SM, Elwazzan VS, Sakran W.  AAPS PharmSciTech. 2021 Jan 6;22(1):33. doi: 10.1208/s12249-020-01893-9.  PMID: 33404930 |
| 50. | [Sertaconazole nitrate loaded nanovesicular systems for targeting skin fungal infection: In-vitro, ex-vivo and in-vivo evaluation.](https://pubmed.ncbi.nlm.nih.gov/28522423/)  Abdellatif MM, Khalil IA, Khalil MAF.  Int J Pharm. 2017 Jul 15;527(1-2):1-11. doi: 10.1016/j.ijpharm.2017.05.029. Epub 2017 May 15.  PMID: 28522423 |
| 51. | [A study of the treatment of cutaneous fungal infection in animal model using photoactivated composite of methylene blue and gold nanoparticle.](https://pubmed.ncbi.nlm.nih.gov/27242275/)  Tawfik AA, Noaman I, El-Elsayyad H, El-Mashad N, Soliman M.  Photodiagnosis Photodyn Ther. 2016 Sep;15:59-69. doi: 10.1016/j.pdpdt.2016.05.010. Epub 2016 May 27.  PMID: 27242275 |
| 52. | [Inhibitory effect of silver nanoparticles mediated by atmospheric pressure air cold plasma jet against dermatophyte fungi.](https://pubmed.ncbi.nlm.nih.gov/26296782/)  Ouf SA, El-Adly AA, Mohamed AH.  J Med Microbiol. 2015 Oct;64(10):1151-1161. doi: 10.1099/jmm.0.000133. Epub 2015 Jul 14.  PMID: 26296782 |
| 53. | [Enhancement of the antidermatophytic activity of silver nanoparticles by Q-switched Nd:YAG laser and monoclonal antibody conjugation.](https://pubmed.ncbi.nlm.nih.gov/28339548/)  Ouf SA, Mohamed AH, El-Adly AA.  Med Mycol. 2017 Jul 1;55(5):495-506. doi: 10.1093/mmy/myw096.  PMID: 28339548 |
| 54. | [Formulation, characterization, and clinical evaluation of microemulsion containing clotrimazole for topical delivery.](https://pubmed.ncbi.nlm.nih.gov/21725708/)  Hashem FM, Shaker DS, Ghorab MK, Nasr M, Ismail A.  AAPS PharmSciTech. 2011 Sep;12(3):879-86. doi: 10.1208/s12249-011-9653-7. Epub 2011 Jul 2.  PMID: 21725708 **Free PMC article.** |
| 55. | [Oil of bitter orange: new topical antifungal agent.](https://pubmed.ncbi.nlm.nih.gov/8737885/)  Ramadan W, Mourad B, Ibrahim S, Sonbol F.  Int J Dermatol. 1996 Jun;35(6):448-9. doi: 10.1111/j.1365-4362.1996.tb03032.x.  PMID: 8737885 Clinical Trial. |
| 56. | [Enhancement of the topical tolnaftate delivery for the treatment of tinea pedis via provesicular gel systems.](https://pubmed.ncbi.nlm.nih.gov/27666873/)  AbouSamra MM, Salama AH.  J Liposome Res. 2017 Dec;27(4):324-334. doi: 10.1080/08982104.2016.1239634. Epub 2016 Oct 19.  PMID: 27666873 |
| 57. | [Onychomycosis in Egypt.](https://pubmed.ncbi.nlm.nih.gov/4249711/)  Abdel-Fattah A, el-Mazny H, Abdullah MA, Refai M.  Mykosen. 1969 Aug 1;12(8):503-6. doi: 10.1111/j.1439-0507.1969.tb03494.x.  PMID: 4249711 No abstract available. |
| 58. | [A comparative clinical and mycological study of Nd-YAG laser versus topical terbinafine in the treatment of onychomycosis.](https://pubmed.ncbi.nlm.nih.gov/25669435/)  El-Tatawy RA, Abd El-Naby NM, El-Hawary EE, Talaat RA.  J Dermatolog Treat. 2015 Oct;26(5):461-4. doi: 10.3109/09546634.2014.998607. Epub 2015 Feb 11.  PMID: 25669435 Clinical Trial. |
| 59. | [Disseminated cryptococcosis with cutaneous lesions.](https://pubmed.ncbi.nlm.nih.gov/8935633/)  Mostafa WZ, Ishak EA, Ekladious EM, Arnaout HH.  J Dermatol. 1996 Mar;23(3):209-13. doi: 10.1111/j.1346-8138.1996.tb03999.x.  PMID: 8935633 |
| 60. | [Importance of mycological confirmation of clinically suspected cases of tinea corporis, tinea pedis and tinea cruris.](https://pubmed.ncbi.nlm.nih.gov/16916049/)  Omar AA.  J Egypt Public Health Assoc. 2004;79(1-2):43-58.  PMID: 16916049 |
| 61. | [Production and evaluation of antimycotic and antihepatitis C virus potential of fusant MERV6270 derived from mangrove endophytic fungi using novel substrates of agroindustrial wastes.](https://pubmed.ncbi.nlm.nih.gov/25234393/)  El-Gendy MM, El-Bondkly AM, Yahya SM.  Appl Biochem Biotechnol. 2014 Dec;174(8):2674-701. doi: 10.1007/s12010-014-1218-2. Epub 2014 Sep 20.  PMID: 25234393 |
| 62. | [Applicability of Fourier transform infrared (FTIR) spectroscopy in rapid identification of some Candida and dermatophyte species infections in humans.](https://pubmed.ncbi.nlm.nih.gov/27337493/)  Mohammed YF, Salem EZ, Shahin IM, Abdo HM, Emam HE, Fawzy M, Abdel Salam MF.  Int J Dermatol. 2016 Oct;55(10):1164-71. doi: 10.1111/ijd.4957. Epub 2016 Jun 23.  PMID: 27337493 |
| 63. | [Dermatophytes and other keratinophilic fungi causing ringworm of horses.](https://pubmed.ncbi.nlm.nih.gov/8919935/)  Mahmoud AL.  Folia Microbiol (Praha). 1995;40(3):293-6. doi: 10.1007/BF02814211.  PMID: 8919935 |
| 64. | [A multicenter clinicomycological study evaluating the spectrum of adult tinea capitis in Egypt.](https://pubmed.ncbi.nlm.nih.gov/24336943/)  El-Khalawany M, Shaaban D, Hassan H, Abdalsalam F, Eassa B, Abdel Kader A, Shaheen I.  Acta Dermatovenerol Alp Pannonica Adriat. 2013 Dec;22(4):77-82.  PMID: 24336943 |
| 65. | [Basic fibroblast growth factor and tumour necrosis factor alpha in vitiligo and other hypopigmented disorders: suggestive possible therapeutic targets.](https://pubmed.ncbi.nlm.nih.gov/22151832/)  Seif El Nasr H, Shaker OG, Fawzi MM, El-Hanafi G.  J Eur Acad Dermatol Venereol. 2013 Jan;27(1):103-8. doi: 10.1111/j.1468-3083.2011.04368.x. Epub 2011 Dec 8.  PMID: 22151832 |
| 66. | [One-week therapy with oral terbinafine in cases of tinea cruris/corporis.](https://pubmed.ncbi.nlm.nih.gov/7999601/)  Farag A, Taha M, Halim S.  Br J Dermatol. 1994 Nov;131(5):684-6. doi: 10.1111/j.1365-2133.1994.tb04983.x.  PMID: 7999601 Clinical Trial. |
| 67. | [Madurella mycetomi as a cause of maduromycosis in Egypt. Clinical and mycopathological studies of two cases.](https://pubmed.ncbi.nlm.nih.gov/4474964/)  Abdei-Aal H, Karim AZ, Moawad MK, Kalifa TM.  Dermatol Monatsschr. 1974 Aug;160(8):670-4.  PMID: 4474964 No abstract available. |
| 68. | [Single-step PCR using (GACA)4 primer: utility for rapid identification of dermatophyte species and strains.](https://pubmed.ncbi.nlm.nih.gov/18579714/)  Shehata AS, Mukherjee PK, Aboulatta HN, el-Akhras AI, Abbadi SH, Ghannoum MA.  J Clin Microbiol. 2008 Aug;46(8):2641-5. doi: 10.1128/JCM.00697-08. Epub 2008 Jun 25.  PMID: 18579714 **Free PMC article.** |
| 69. | [Diagnostic performance of molecular and conventional methods for identification of dermatophyte species from clinically infected Arabian horses in Egypt.](https://pubmed.ncbi.nlm.nih.gov/27549079/)  Tartor YH, El Damaty HM, Mahmmod YS.  Vet Dermatol. 2016 Oct;27(5):401-e102. doi: 10.1111/vde.12372. Epub 2016 Aug 22.  PMID: 27549079 |
| 70. | [Physically cross-linked polyvinyl alcohol for the topical delivery of fluconazole.](https://pubmed.ncbi.nlm.nih.gov/18798033/)  Abdel-Mottaleb MM, Mortada ND, El-Shamy AA, Awad GA.  Drug Dev Ind Pharm. 2009 Mar;35(3):311-20. doi: 10.1080/03639040802325893.  PMID: 18798033 |
| 71. | [Could nanovesicles containing a penetration enhancer clinically improve the therapeutic outcome in skin fungal diseases?](https://pubmed.ncbi.nlm.nih.gov/26135513/)  Bsieso EA, Nasr M, Moftah NH, Sammour OA, Abd El Gawad NA.  Nanomedicine (Lond). 2015;10(13):2017-31. doi: 10.2217/nnm.15.49. Epub 2015 Jul 2.  PMID: 26135513 |
| 72. | [Skin granuloma in the Nile Valley.](https://pubmed.ncbi.nlm.nih.gov/5425322/)  el Mofty AM, Nada MM.  Int J Dermatol. 1970 Jan-Mar;9(1):33-40. doi: 10.1111/j.1365-4362.1970.tb04725.x.  PMID: 5425322 No abstract available. |
| 73. | [Epidemiological trends of superficial fungal infections in Upper Egypt: a cohort observational study.](https://pubmed.ncbi.nlm.nih.gov/29952296/)  Saleh R, Abd Elmaged WM, El-Saied DA.  Eur J Dermatol. 2018 Aug 1;28(4):528-530. doi: 10.1684/ejd.2018.3307.  PMID: 29952296 No abstract available. |
| 74. | [Tinea versicolor of the scalp.](https://pubmed.ncbi.nlm.nih.gov/7591430/)  el-Gothamy Z, Ghozzi M.  Int J Dermatol. 1995 Aug;34(8):533-4. doi: 10.1111/j.1365-4362.1995.tb02946.x.  PMID: 7591430 |
| 75. | [Clinical study of a new preparation of 8-methoxypsoralen in photochemotherapy.](https://pubmed.ncbi.nlm.nih.gov/7960360/)  el-Mofty AM, el-Sawalhy H, el-Mofty M.  Int J Dermatol. 1994 Aug;33(8):588-92. doi: 10.1111/j.1365-4362.1994.tb02904.x.  PMID: 7960360 Clinical Trial. |
| 76. | [Coadministration of ketoconazole to cyclosporin-treated kidney transplant recipients: a prospective randomized study.](https://pubmed.ncbi.nlm.nih.gov/8546171/)  Sobh M, el-Agroudy A, Moustafa F, Harras F, el-Bedewy M, Ghoneim M.  Am J Nephrol. 1995;15(6):493-9. doi: 10.1159/000168892.  PMID: 8546171 Clinical Trial. |
| 77. | [Nail dermoscopy is a helpful tool in the diagnosis of onychomycosis: A case control study.](https://pubmed.ncbi.nlm.nih.gov/26576042/)  El-Hoshy KH, Abdel Hay RM, El-Sherif RH, Salah Eldin M, Moussa MF.  Eur J Dermatol. 2015 Sep-Oct;25(5):494-5. doi: 10.1684/ejd.2015.2637.  PMID: 26576042 No abstract available. |
| 78. | [Invasive zygomycosis with a fatal outcome.](https://pubmed.ncbi.nlm.nih.gov/16230557/)  Abdel-Naser MB, Yousef N, el-Fakar NZ, Abdullatif OH, Wollina U, Abdallah MA.  Arch Dermatol. 2005 Oct;141(10):1211-3. doi: 10.1001/archderm.141.10.1211.  PMID: 16230557 No abstract available. |
| 79. | [Animal ringworm in upper Egypt.](https://pubmed.ncbi.nlm.nih.gov/1265571/)  Abou-Gabal M, El-Galil GA, El-Nor EA, El-Rehim DA.  Sabouraudia. 1976 Mar;14(1):33-6. doi: 10.1080/00362177685190061.  PMID: 1265571 |
| 80. | [Laboratory acquired infection with Keratinomyces ajelloi.](https://pubmed.ncbi.nlm.nih.gov/5464089/)  Refai M, Ali AH.  Mykosen. 1970 Jun 1;13(6):317-8. doi: 10.1111/j.1439-0507.1970.tb01277.x.  PMID: 5464089 No abstract available. |
| 81. | [Some deep mycoses prevalent in the United Arab Republic.](https://pubmed.ncbi.nlm.nih.gov/4497480/)  el-Zawahry M.  Int J Dermatol. 1974 Jul-Aug;13(4):210-4. doi: 10.1111/j.1365-4362.1974.tb01795.x.  PMID: 4497480 No abstract available. |
| 82. | [A study of dermatophytoses in Sana'a, Yemen Republic.](https://pubmed.ncbi.nlm.nih.gov/12000511/)  Mahmoud AL.  Mycoses. 2002 Apr;45(3-4):105-8. doi: 10.1046/j.1439-0507.2002.00729.x.  PMID: 12000511 |
| 83. | [Progressive cutaneous Cryptococcosis complicated with meningitis in a myasthenia gravis patient on long-term immunosuppressive therapy - a case report.](https://pubmed.ncbi.nlm.nih.gov/28446137/)  Huong NTC, Altibi AMA, Hoa NM, Tuan LA, Salman S, Morsy S, Lien NTB, Truong NT, Mai NTH, Hoa PTL, Thang NB, Trung VT.  BMC Infect Dis. 2017 Apr 26;17(1):311. doi: 10.1186/s12879-017-2415-8.  PMID: 28446137 **Free PMC article.** |
| 84. | [Chronic mucocutaneous candidiasis associated with an SH2 domain gain-of-function mutation that enhances STAT1 phosphorylation.](https://pubmed.ncbi.nlm.nih.gov/26948078/)  Sobh A, Chou J, Schneider L, Geha RS, Massaad MJ.  J Allergy Clin Immunol. 2016 Jul;138(1):297-299. doi: 10.1016/j.jaci.2015.12.1320. Epub 2016 Mar 2.  PMID: 26948078 No abstract available. |
| 85. | [Identification of three yeast species using the conventional and internal transcribed spacer region sequencing methods as first or second global record from human superficial infections.](https://pubmed.ncbi.nlm.nih.gov/27392537/)  Abdel-Sater MA, Moubasher AA, Soliman Z.  Mycoses. 2016 Oct;59(10):652-61. doi: 10.1111/myc.12520. Epub 2016 Jul 9.  PMID: 27392537 |
| 86. | [Naftifine versus miconazole/hydrocortisone in inflammatory dermatophyte infections.](https://pubmed.ncbi.nlm.nih.gov/7960355/)  Nada M, Hanafi S, al-Omari H, Mokhtar M, el-Shamy S, Mühlbacher J.  Int J Dermatol. 1994 Aug;33(8):570-2. doi: 10.1111/j.1365-4362.1994.tb02899.x.  PMID: 7960355 Clinical Trial. No abstract available. |
| 87. | [Ringworm of the scalp in primary-school children in Alexandria: infection and carriage.](https://pubmed.ncbi.nlm.nih.gov/12197355/)  Omar AA.  East Mediterr Health J. 2000 Sep-Nov;6(5-6):961-7.  PMID: 12197355 |
| 88. | [Incidence of dermatophytes and cyclohexamide resistant fungi on healthy children hairs and nails in nurseries.](https://pubmed.ncbi.nlm.nih.gov/12206316/)  Maghazy SM.  Mycopathologia. 2002;154(4):171-5. doi: 10.1023/a:1016381629089.  PMID: 12206316 |
| 89. | [A pigment-producing dermatophyte difficult to classify as Trichophyton rubrum or Trichophyton violaceum isolated from cases of tinea cruris and tinea corporis in Egypt.](https://pubmed.ncbi.nlm.nih.gov/4564495/)  Abdallah MA, Refai M, Rieth H.  Mykosen. 1972 Jun 1;15(6):245-8. doi: 10.1111/j.1439-0507.1972.tb02502.x.  PMID: 4564495 No abstract available. |
| 90. | [Topically applied griseofulvin in the treatment of superficial dermatomycoses in Egypt.](https://pubmed.ncbi.nlm.nih.gov/913867/)  Abdel-Aal H, EL-Shazli M, Saleh AM.  J Int Med Res. 1977;5(5):382-6. doi: 10.1177/030006057700500517.  PMID: 913867 |
| 91. | [Trichophyton rubrum infection in a family transmitted from a cat.](https://pubmed.ncbi.nlm.nih.gov/5761179/)  Refai M, Miligy M.  Mykosen. 1968 Mar 1;11(3):191-4. doi: 10.1111/j.1439-0507.1968.tb03320.x.  PMID: 5761179 No abstract available. |
| 92. | [Studies on pityriasis versicolor in Egypt. II. Clinical and therapeutic.](https://pubmed.ncbi.nlm.nih.gov/5110393/)  el-Hefnawi H, el-Gothamy Z, Refai M.  Mykosen. 1971 Oct 1;14(10):483-90. doi: 10.1111/j.1439-0507.1971.tb02936.x.  PMID: 5110393 No abstract available. |
| 93. | [A survey of histoplasmosis and blastomycosis in U.A.R. (Egyptian sector). A preliminary report.](https://pubmed.ncbi.nlm.nih.gov/5796000/)  el-Mofty AM, Mikhail GR, Nada MM, Moawad MK.  Mycopathol Mycol Appl. 1969 May 23;37(3):257-62. doi: 10.1007/BF02051359.  PMID: 5796000 No abstract available. |
| 94. | [The perfect state of Trichophyton violaceum.](https://pubmed.ncbi.nlm.nih.gov/7928033/)  Taha M, Amer M, Salem A, el Harras M.  Int J Dermatol. 1994 Jul;33(7):493-5. doi: 10.1111/j.1365-4362.1994.tb02862.x.  PMID: 7928033 |
| 95. | [[NOTES ON DERMATOSES IN EGYPT].](https://pubmed.ncbi.nlm.nih.gov/14127037/)  ZAWAHRYMM EL.  Maroc Med. 1964 Jan;43:46-53.  PMID: 14127037 French. No abstract available. |
| 96. | [ONYCHOMYCOSIS GENERAL REVIEW AND TREATMENT.](https://pubmed.ncbi.nlm.nih.gov/14162103/)  ELZAWAHRY M.  J Egypt Med Assoc. 1963;46:1267-9.  PMID: 14162103 No abstract available. |
| 97. | [The frequency of causative dermatophytes in Egypt.](https://pubmed.ncbi.nlm.nih.gov/7263125/)  Amer M, Taha M, Tosson Z, El-Garf A.  Int J Dermatol. 1981 Jul-Aug;20(6):431-4. doi: 10.1111/j.1365-4362.1981.tb02009.x.  PMID: 7263125 No abstract available. |
| 98. | [[The enzymatic activity of fungi strains isolated from the skin and skin appendages of humans returning from the tropics].](https://pubmed.ncbi.nlm.nih.gov/16886418/)  Głowacka A, Ochecka-Szymańska A.  Wiad Parazytol. 2001;47(4):729-33.  PMID: 16886418 Polish. |
| 99. | [ERYTHEMA ANNULARE CENTRIFUGUM AS A DERMATOPHYTID.](https://pubmed.ncbi.nlm.nih.gov/14162117/)  ELMOFTY AM, NADA MM.  J Egypt Med Assoc. 1963;46:979-84.  PMID: 14162117 No abstract available. |
| 100. | [Class-specific antibody in human dermatophytosis reactive with Trichophyton rubrum derived antigen.](https://pubmed.ncbi.nlm.nih.gov/7984217/)  Hamouda T, Jeffries CD, Ekladios EM, el-Mishad AM, el-Koomy M, Saleh N.  Mycopathologia. 1994 Aug;127(2):83-8. doi: 10.1007/BF01103063.  PMID: 7984217 |
| 101. | [Dermatophytes and other associated fungi isolated from ringworm lesions of camels.](https://pubmed.ncbi.nlm.nih.gov/8150398/)  Mahmoud AL.  Folia Microbiol (Praha). 1993;38(6):505-8. doi: 10.1007/BF02814404.  PMID: 8150398 |
| 102. | [SPOROTRICHOSIS IN EGYPT.](https://pubmed.ncbi.nlm.nih.gov/14314418/)  EL-MOFTY AM, NADA M.  Br J Dermatol. 1965 Jul;77:357-64. doi: 10.1111/j.1365-2133.1965.tb14661.x.  PMID: 14314418 No abstract available. |
| 103. | [The radiological picture of mycetoma; report of a case verified by biopsy.](https://pubmed.ncbi.nlm.nih.gov/18143687/)  EL DINE MASSOUD G.  J Egypt Med Assoc. 1949 Jan;32(1):64-8.  PMID: 18143687 No abstract available. |
| 104. | [Fluconazole in the treatment of tinea versicolor. Egyptian Fluconazole Study Group.](https://pubmed.ncbi.nlm.nih.gov/9466206/)  Amer MA.  Int J Dermatol. 1997 Dec;36(12):940-2. doi: 10.1046/j.1365-4362.1997.00213.x.  PMID: 9466206 Clinical Trial. No abstract available. |
| 105. | [Tinea capitis in Egypt.](https://pubmed.ncbi.nlm.nih.gov/5595806/)  Abdel Fattah A, El-Gothamy Z.  Mykosen. 1967 May 1;10(5):189-94. doi: 10.1111/j.1439-0507.1967.tb02850.x.  PMID: 5595806 No abstract available. |
| 106. | [Studies on pityriasis versicolor in Egypt. I. Incidence.](https://pubmed.ncbi.nlm.nih.gov/5575840/)  el-Hefnawi H, el-Gothamy Z, Refai M.  Mykosen. 1971 May 1;14(5):225-31. doi: 10.1111/j.1439-0507.1971.tb03041.x.  PMID: 5575840 No abstract available. |
| 107. | [Clinical experience with Canesten-cream (Bay b 5097) against dermatophytes in Egypt.](https://pubmed.ncbi.nlm.nih.gov/4771479/)  Aal HA, Moawad MK.  Mykosen. 1973 Nov 1;16(11):369-71. doi: 10.1111/j.1439-0507.1973.tb04075.x.  PMID: 4771479 No abstract available. |
| 108. | [Primary cutaneous cryptococcosis in Egypt.](https://pubmed.ncbi.nlm.nih.gov/1176246/)  Abdel-Fattah A, Zeid MS, Ghaly AF.  Int J Dermatol. 1975 Oct;14(8):606-9. doi: 10.1111/j.1365-4362.1975.tb00149.x.  PMID: 1176246 |
| 109. | [[Tinea of the scalp in Egypt].](https://pubmed.ncbi.nlm.nih.gov/6872104/)  Othman T, Vacher C.  Bull Soc Pathol Exot Filiales. 1983 Mar-Apr;76(2):126-8.  PMID: 6872104 French. |
| 110. | [Clinical diagnosis of T. capitis.](https://pubmed.ncbi.nlm.nih.gov/13321236/)  GALLOWAY CB.  Med J Egypt Armed Forces. 1956 Mar;2(1):33-6.  PMID: 13321236 No abstract available. |
| 111. | [[Fungal diseases among the people and animals of Egypt].](https://pubmed.ncbi.nlm.nih.gov/997879/)  Refai M.  Vestn Dermatol Venerol. 1976 Jul;(7):36-40.  PMID: 997879 Russian. No abstract available. |
| 112. | [Incidence of fungus infection in U.A.R.](https://pubmed.ncbi.nlm.nih.gov/13993651/)  EL-ZAWAHRY M.  J Egypt Med Assoc. 1963;46:78-81.  PMID: 13993651 No abstract available. |
| 113. | [Is thallium epilation dangerous in childhood?](https://pubmed.ncbi.nlm.nih.gov/13070299/)  KENEDY DA.  J Egypt Med Assoc. 1953;36(1):59-68.  PMID: 13070299 No abstract available. |
| 114. | [INCIDENCE OF FUNGUS INFECTION IN U.A.R.](https://pubmed.ncbi.nlm.nih.gov/14080707/)  ZAWAHRYM EL.  Indian J Dermatol. 1963 Oct;9:9-10.  PMID: 14080707 No abstract available. |
| 115. | [NOTES CONCERNING DERMATOSES IN OUR COUNTRIES.](https://pubmed.ncbi.nlm.nih.gov/14162097/)  EL-ZAWAHRY M.  J Egypt Med Assoc. 1963;46:1155-62.  PMID: 14162097 No abstract available. |
| 116. | [TREATMENT OF EPIDERMAL MYCOSES WITH ASTEROL.](https://pubmed.ncbi.nlm.nih.gov/14162112/)  MALEK AI.  J Egypt Med Assoc. 1963;46:1357-60.  PMID: 14162112 No abstract available. |
| 117. | [Treatment of fungus infections with griseofulvin.](https://pubmed.ncbi.nlm.nih.gov/14461148/)  EL-MOFTY AM.  J Egypt Med Assoc. 1961;44:772-87.  PMID: 14461148 No abstract available. |
| 118. | [Study of tinea cruris in Egypt.](https://pubmed.ncbi.nlm.nih.gov/4636066/)  el-Mazny H, Abdel-Fattah A, Abdallah MA, Refai M.  Mykosen. 1972 Aug 1;15(8):331-5. doi: 10.1111/j.1439-0507.1972.tb02526.x.  PMID: 4636066 No abstract available. |
| 119. | [Study of tinea pedis in Egypt.](https://pubmed.ncbi.nlm.nih.gov/13752862/)  KHAFAGY HM.  J Egypt Med Assoc. 1960;43:489-90.  PMID: 13752862 No abstract available. |
| 120. | [Studies on pityriasis versicolor in Egypt. 3. Laboratory diagnosis and experimental infection.](https://pubmed.ncbi.nlm.nih.gov/5075545/)  el-Hefnawi H, el-Gothamy Z, Refai M.  Mykosen. 1972 Apr;15(4):165-70. doi: 10.1111/j.1439-0507.1972.tb02468.x.  PMID: 5075545 No abstract available. |
| 121. | [NOTES CONCERNING FUNGUS INFECTION IN U.A.R.](https://pubmed.ncbi.nlm.nih.gov/14162090/)  EL-ZAWAHRY M.  J Egypt Med Assoc. 1963;46:1051-65.  PMID: 14162090 No abstract available. |
| 122. | [Clinical and mycological study of the macerated toe web in Egypt.](https://pubmed.ncbi.nlm.nih.gov/5548033/)  Abd-Allah MA, Abdel-Fattah A, el Mazny, Refai M.  Mykosen. 1971 Feb 1;14(2):83-8. doi: 10.1111/j.1439-0507.1971.tb02993.x.  PMID: 5548033 No abstract available. |
| 123. | [Ringworm in animals in a farm in Assiut.](https://pubmed.ncbi.nlm.nih.gov/5102750/)  Abdallah IS, Gelil GA, Hamid YM, Refai M.  Mykosen. 1971 Apr 1;14(4):175-8. doi: 10.1111/j.1439-0507.1971.tb03024.x.  PMID: 5102750 No abstract available. |
| 124. | [Ring worm of scalp in Egyptian children.](https://pubmed.ncbi.nlm.nih.gov/14928252/)  EL-MOSLEMANI AA.  J Egypt Med Assoc. 1951;34(6):409-10.  PMID: 14928252 No abstract available. |
| 125. | [[Contribution to the dermatophyte flora of Northern Egypt].](https://pubmed.ncbi.nlm.nih.gov/5595373/)  Refai M.  Mykosen. 1967 Feb 1;10(2):61-2.  PMID: 5595373 German. No abstract available. |
| 126. | [Oral and cutaneous candidosis in Saudi Arabian newborn infants.](https://pubmed.ncbi.nlm.nih.gov/6948679/)  Sadek S, Mostafa MH, Radwan Z.  Egypt Dent J. 1980 Jan;26(1):73-86.  PMID: 6948679 No abstract available. |
| 127. | [TINEA CAPITIS: A STUDY OF 400 EGYPTIAN CASES.](https://pubmed.ncbi.nlm.nih.gov/14176377/)  TAYLOR WW Jr, BASSALY M.  J Trop Med Hyg. 1964 Jul;67:180-2.  PMID: 14176377 No abstract available. |
| 128. | [A survey of the pathogenic fungi of mycotic infection of the scalp in U.A.R.](https://pubmed.ncbi.nlm.nih.gov/5650626/)  El Mofty AM, Jeffries CD, El Komy HM.  Mycopathol Mycol Appl. 1968 Jan 19;34(1):61-4. doi: 10.1007/BF02050846.  PMID: 5650626 No abstract available. |
| 129. | [Cattie ringworm caused by Trichophyton verrucosum as a source of tinea capitis in children.](https://pubmed.ncbi.nlm.nih.gov/13819849/)  EL-FIKI AY, RIETH H.  J Egypt Med Assoc. 1959;42:540-3.  PMID: 13819849 No abstract available. |
| 130. | [Tinea capitis; a mycological study of three hundred and three cases.](https://pubmed.ncbi.nlm.nih.gov/13184069/)  MIKHAIL GR.  J Egypt Med Assoc. 1954 Jun;37(5):538-54.  PMID: 13184069 No abstract available. |
| 131. | [[Occurrence of Trichophyton verrucosum infections in Egypt, with suggestions for treatment using griseofulvin].](https://pubmed.ncbi.nlm.nih.gov/765093/)  Refai M, Ibrahim MS, Ei-Saifi A.  Dtsch Tierarztl Wochenschr. 1976 Feb 5;83(2):62-4.  PMID: 765093 German. No abstract available. |

- Search results *after applying the “Human” filter* to the above search

**Search: (((((((((((((Mycetoma[MeSH Major Topic]) OR (Actinomycetoma[MeSH Terms])) OR (Eumycetoma[MeSH Terms])) OR (Madura Foot[MeSH Terms])) OR (Maduromycosis[MeSH Terms])) OR ("actinomycetoma"[All Fields])) OR ("eumycetoma"[All Fields])) OR ("madura foot"[All Fields])) OR ("maduromycosis"[All Fields])) OR ("mycetoma"[All Fields])) AND (Egypt) ("mycetoma"[MeSH Major Topic] OR "mycetoma"[MeSH Terms] OR "mycetoma"[MeSH Terms] OR "mycetoma"[MeSH Terms] OR "mycetoma"[MeSH Terms] OR "actinomycetoma"[All Fields] OR "eumycetoma"[All Fields] OR "madura foot"[All Fields] OR "maduromycosis"[All Fields] OR "mycetoma"[All Fields]) ) OR (Dermatomycoses[MeSH Major Topic])) OR ("dermatomycoses"[All Fields])) AND ("egypt"[All Fields]) Filters: Humans**

Items 1-115 of 115 ([Display the 115 citations in PubMed](https://pubmed.ncbi.nlm.nih.gov/?term=26692117,31633447,29239242,32304603,20718613,31502751,31728627,28327256,14314419,22575159,25330098,30946748,31157575,26261140,32667111,32182387,31025207,30775844,28188051,31493312,32975877,20849434,25481439,32266837,31511056,30081698,26707860,33141453,19767633,26447337,26413063,18972221,21506964,25953414,29314351,32162164,29768066,23760076,12916729,27654953,16681816,27264960,31697010,33404930,27242275,26296782,28339548,21725708,8737885,27666873,4249711,25669435,8935633,16916049,27337493,24336943,22151832,7999601,4474964,18579714,26135513,5425322,29952296,7591430,7960360,8546171,26576042,16230557,1265571,5464089,4497480,12000511,28446137,26948078,27392537,7960355,12197355,12206316,913867,5761179,5110393,5796000,7928033,14127037,14162103,7263125,16886418,14162117,7984217,14314418,18143687,9466206,5595806,5575840,4771479,1176246,6872104,997879,14080707,14162097,14162112,13993651,13070299,13752862,4636066,5075545,14162090,5548033,5102750,5595373,6948679,14176377,5650626,13819849,13184069))

| 1. | [Dermatophytosis in northern Africa.](https://pubmed.ncbi.nlm.nih.gov/26692117/)  Nweze EI, Eke I.  Mycoses. 2016 Mar;59(3):137-44. doi: 10.1111/myc.12447. Epub 2015 Dec 22.  PMID: 26692117 Review. |
| --- | --- |
| 2. | [The potential role of interleukin-37 in infectious diseases.](https://pubmed.ncbi.nlm.nih.gov/31633447/)  Allam G, Gaber AM, Othman SI, Abdel-Moneim A.  Int Rev Immunol. 2020;39(1):3-10. doi: 10.1080/08830185.2019.1677644. Epub 2019 Oct 21.  PMID: 31633447 Review. |
| 3. | [Fluconazole-loaded solid lipid nanoparticles topical gel for treatment of pityriasis versicolor: formulation and clinical study.](https://pubmed.ncbi.nlm.nih.gov/29239242/)  El-Housiny S, Shams Eldeen MA, El-Attar YA, Salem HA, Attia D, Bendas ER, El-Nabarawi MA.  Drug Deliv. 2018 Nov;25(1):78-90. doi: 10.1080/10717544.2017.1413444.  PMID: 29239242 **Free PMC article.** Clinical Trial. |
| 4. | [In vitro antifungal susceptibility testing of fungi in patients with onychomycosis.](https://pubmed.ncbi.nlm.nih.gov/32304603/)  Abu El-Hamd M, Abd Elhameed MI, Shalaby MFM, Saleh R.  Dermatol Ther. 2020 May;33(3):e13429. doi: 10.1111/dth.13429. Epub 2020 May 8.  PMID: 32304603 |
| 5. | [Invasive aspergillosis in developing countries.](https://pubmed.ncbi.nlm.nih.gov/20718613/)  Chakrabarti A, Chatterjee SS, Das A, Shivaprakash MR.  Med Mycol. 2011 Apr;49 Suppl 1:S35-47. doi: 10.3109/13693786.2010.505206. Epub 2010 Aug 18.  PMID: 20718613 Review. |
| 6. | [Onychomycosis: Correlation between the dermoscopic patterns and fungal culture.](https://pubmed.ncbi.nlm.nih.gov/31502751/)  Abdallah NA, Said M, Mahmoud MT, Omar MA.  J Cosmet Dermatol. 2020 May;19(5):1196-1204. doi: 10.1111/jocd.13144. Epub 2019 Sep 10.  PMID: 31502751 |
| 7. | [Diagnosis of onychomycosis clinically by nail dermoscopy versus microbiological diagnosis.](https://pubmed.ncbi.nlm.nih.gov/31728627/)  Nada EEA, El Taieb MA, El-Feky MA, Ibrahim HM, Hegazy EM, Mohamed AE, El-Amir MI.  Arch Dermatol Res. 2020 Apr;312(3):207-212. doi: 10.1007/s00403-019-02008-6. Epub 2019 Nov 14.  PMID: 31728627 |
| 8. | [Origin and distribution of Sporothrix globosa causing sapronoses in Asia.](https://pubmed.ncbi.nlm.nih.gov/28327256/)  Moussa TAA, Kadasa NMS, Al Zahrani HS, Ahmed SA, Feng P, Gerrits van den Ende AHG, Zhang Y, Kano R, Li F, Li S, Song Y, Dong B, Rossato L, Dolatabadi S, Hoog S.  J Med Microbiol. 2017 May;66(5):560-569. doi: 10.1099/jmm.0.000451. Epub 2017 May 22.  PMID: 28327256 Review. |
| 9. | [MADURA FOOT" IN EGYPT.](https://pubmed.ncbi.nlm.nih.gov/14314419/)  EL-MOFTY AM, ISKANDER IO, NADA M, ZAKI SM.  Br J Dermatol. 1965 Jul;77:365-72. doi: 10.1111/j.1365-2133.1965.tb14662.x.  PMID: 14314419 No abstract available. |
| 10. | [Hair loss in pityriasis versicolor lesions: a descriptive clinicopathological study.](https://pubmed.ncbi.nlm.nih.gov/22575159/)  Mostafa WZ, Assaf MI, Ameen IA, El Safoury OS, Al Sulh SA.  J Am Acad Dermatol. 2013 Jul;69(1):e19-23. doi: 10.1016/j.jaad.2012.03.004. Epub 2012 May 8.  PMID: 22575159 |
| 11. | [Mapping the potential risk of mycetoma infection in Sudan and South Sudan using ecological niche modeling.](https://pubmed.ncbi.nlm.nih.gov/25330098/)  Samy AM, van de Sande WW, Fahal AH, Peterson AT.  PLoS Negl Trop Dis. 2014 Oct 16;8(10):e3250. doi: 10.1371/journal.pntd.0003250. eCollection 2014 Oct.  PMID: 25330098 **Free PMC article.** |
| 12. | [The Role of Interleukin-1 cytokine family (IL-1β, IL-37) and interleukin-12 cytokine family (IL-12, IL-35) in eumycetoma infection pathogenesis.](https://pubmed.ncbi.nlm.nih.gov/30946748/)  Abushouk A, Nasr A, Masuadi E, Allam G, Siddig EE, Fahal AH.  PLoS Negl Trop Dis. 2019 Apr 4;13(4):e0007098. doi: 10.1371/journal.pntd.0007098. eCollection 2019 Apr.  PMID: 30946748 **Free PMC article.** |
| 13. | [Combined long-pulsed Nd-Yag laser and itraconazole versus itraconazole alone in the treatment of onychomycosis nails.](https://pubmed.ncbi.nlm.nih.gov/31157575/)  Hamed Khater M, Khattab FM.  J Dermatolog Treat. 2020 Jun;31(4):406-409. doi: 10.1080/09546634.2019.1623861. Epub 2019 Jun 14.  PMID: 31157575 Clinical Trial. |
| 14. | [Recent advances in topical formulation carriers of antifungal agents.](https://pubmed.ncbi.nlm.nih.gov/26261140/)  Bseiso EA, Nasr M, Sammour O, Abd El Gawad NA.  Indian J Dermatol Venereol Leprol. 2015 Sep-Oct;81(5):457-63. doi: 10.4103/0378-6323.162328.  PMID: 26261140 Review. |
| 15. | [Successful treatment of resistant onychomycosis with voriconazole in a liver transplant patient.](https://pubmed.ncbi.nlm.nih.gov/32667111/)  Nofal A, Fawzy MM, El-Hawary EE.  Dermatol Ther. 2020 Nov;33(6):e14014. doi: 10.1111/dth.14014. Epub 2020 Aug 12.  PMID: 32667111 |
| 16. | [Adapalene gel 0.1% vs ketoconazole cream 2% and their combination in treatment of pityriasis versicolor: A randomized clinical study.](https://pubmed.ncbi.nlm.nih.gov/32182387/)  Bakr E, Abdo H, Abd-Elaziz H, Abd-Elrazek H, Amer M.  Dermatol Ther. 2020 May;33(3):e13319. doi: 10.1111/dth.13319. Epub 2020 Mar 30.  PMID: 32182387 Clinical Trial. |
| 17. | [Fractional carbon dioxide laser and topical tioconazole in the treatment of fingernail onychomycosis.](https://pubmed.ncbi.nlm.nih.gov/31025207/)  El-Tatawy RA, Aliweh HA, Hegab DS, Talaat RAZ, Shams Eldeen MA.  Lasers Med Sci. 2019 Dec;34(9):1873-1880. doi: 10.1007/s10103-019-02789-2. Epub 2019 Apr 25.  PMID: 31025207 |
| 18. | [Trichophyton mentagrophytes - a new genotype of zoophilic dermatophyte causes sexually transmitted infections.](https://pubmed.ncbi.nlm.nih.gov/30775844/)  Kupsch C, Czaika VA, Deutsch C, Gräser Y.  J Dtsch Dermatol Ges. 2019 May;17(5):493-501. doi: 10.1111/ddg.13776. Epub 2019 Feb 18.  PMID: 30775844 |
| 19. | [Incidence and biodiversity of yeasts, dermatophytes and non-dermatophytes in superficial skin infections in Assiut, Egypt.](https://pubmed.ncbi.nlm.nih.gov/28188051/)  Moubasher AH, Abdel-Sater MA, Soliman Z.  J Mycol Med. 2017 Jun;27(2):166-179. doi: 10.1016/j.mycmed.2017.01.005. Epub 2017 Feb 7.  PMID: 28188051 |
| 20. | [Towards a rapid identification and a novel proteomic analysis for dermatophytes from human and animal dermatophytosis.](https://pubmed.ncbi.nlm.nih.gov/31493312/)  Tartor YH, Abo Hashem ME, Enany S.  Mycoses. 2019 Dec;62(12):1116-1126. doi: 10.1111/myc.12998. Epub 2019 Oct 16.  PMID: 31493312 |
| 21. | [Comparative study between the effect of topical tazarotene 0.1 gel alone vs its combination with tioconazole nail paint in treatment of onychomycosis.](https://pubmed.ncbi.nlm.nih.gov/32975877/)  El-Salam SSA, Omar GA, Mahmoud MT, Said M.  Dermatol Ther. 2020 Nov;33(6):e14333. doi: 10.1111/dth.14333. Epub 2020 Oct 9.  PMID: 32975877 |
| 22. | [Skin infections in Egyptian renal transplant recipients.](https://pubmed.ncbi.nlm.nih.gov/20849434/)  Bakr NI, El-Sawy E, Hamdy AF, Bakr MA.  Transpl Infect Dis. 2011 Apr;13(2):131-5. doi: 10.1111/j.1399-3062.2010.00568.x. Epub 2010 Sep 16.  PMID: 20849434 |
| 23. | [Development of topical therapeutics for management of onychomycosis and other nail disorders: a pharmaceutical perspective.](https://pubmed.ncbi.nlm.nih.gov/25481439/)  Elsayed MM.  J Control Release. 2015 Feb 10;199:132-44. doi: 10.1016/j.jconrel.2014.11.017. Epub 2014 Dec 4.  PMID: 25481439 Review. |
| 24. | [Oxiconazole nitrate solid lipid nanoparticles: formulation, in-vitro characterization and clinical assessment of an analogous loaded carbopol gel.](https://pubmed.ncbi.nlm.nih.gov/32266837/)  Mahmoud RA, Hussein AK, Nasef GA, Mansour HF.  Drug Dev Ind Pharm. 2020 May;46(5):706-716. doi: 10.1080/03639045.2020.1752707. Epub 2020 Apr 15.  PMID: 32266837 Clinical Trial. |
| 25. | [Identifying asymptomatic Leishmania infections in non-endemic villages in Gedaref state, Sudan.](https://pubmed.ncbi.nlm.nih.gov/31511056/)  Mohamed NS, Osman HA, Muneer MS, Samy AM, Ahmed A, Mohammed AO, Siddig EE, Abdel Hamid MM, Ali MS, Omer RA, Elaagip AH.  BMC Res Notes. 2019 Sep 11;12(1):566. doi: 10.1186/s13104-019-4608-2.  PMID: 31511056 **Free PMC article.** |
| 26. | [Fractional carbon dioxide laser assisted delivery of topical tazarotene versus topical tioconazole in the treatment of onychomycosis.](https://pubmed.ncbi.nlm.nih.gov/30081698/)  Abd El-Aal EB, Abdo HM, Ibrahim SM, Eldestawy MT.  J Dermatolog Treat. 2019 May;30(3):277-282. doi: 10.1080/09546634.2018.1509046. Epub 2018 Sep 28.  PMID: 30081698 Clinical Trial. |
| 27. | [Control of tinea capitis.](https://pubmed.ncbi.nlm.nih.gov/26707860/)  Gohar A.  Br J Dermatol. 2016 May;174(5):1158. doi: 10.1111/bjd.14372. Epub 2016 Mar 14.  PMID: 26707860 No abstract available. |
| 28. | [Trichoscopic signs of tinea capitis: a guide for selection of appropriate antifungal.](https://pubmed.ncbi.nlm.nih.gov/33141453/)  Genedy RM, Sorour OA, Elokazy MAW.  Int J Dermatol. 2021 Apr;60(4):471-481. doi: 10.1111/ijd.15289. Epub 2020 Nov 3.  PMID: 33141453 |
| 29. | [Nail changes in kidney transplant recipients.](https://pubmed.ncbi.nlm.nih.gov/19767633/)  Abdelaziz AM, Mahmoud KM, Elsawy EM, Bakr MA.  Nephrol Dial Transplant. 2010 Jan;25(1):274-7. doi: 10.1093/ndt/gfp486. Epub 2009 Sep 19.  PMID: 19767633 |
| 30. | [Novel nail penetration enhancer containing vesicles "nPEVs" for treatment of onychomycosis.](https://pubmed.ncbi.nlm.nih.gov/26447337/)  Bseiso EA, Nasr M, Sammour OA, Abd El Gawad NA.  Drug Deliv. 2016 Oct;23(8):2813-2819. doi: 10.3109/10717544.2015.1099059. Epub 2015 Oct 8.  PMID: 26447337 |
| 31. | [Dermatophytes and other associated fungi in patients attending to some hospitals in Egypt.](https://pubmed.ncbi.nlm.nih.gov/26413063/)  Abd Elmegeed AS, Ouf SA, Moussa TA, Eltahlawi SM.  Braz J Microbiol. 2015 Jul 1;46(3):799-805. doi: 10.1590/S1517-838246320140615. eCollection 2015 Jul-Sep.  PMID: 26413063 **Free PMC article.** |
| 32. | [Dermatophyte infections in Cairo, Egypt.](https://pubmed.ncbi.nlm.nih.gov/18972221/)  Zaki SM, Ibrahim N, Aoyama K, Shetaia YM, Abdel-Ghany K, Mikami Y.  Mycopathologia. 2009 Mar;167(3):133-7. doi: 10.1007/s11046-008-9165-5. Epub 2008 Oct 30.  PMID: 18972221 |
| 33. | [Cowden's syndrome: a clinical, immunological, and histopathological study.](https://pubmed.ncbi.nlm.nih.gov/21506964/)  Amer M, Mostafa FF, Attwa EM, Ibrahim S.  Int J Dermatol. 2011 May;50(5):516-21. doi: 10.1111/j.1365-4632.2010.04669.x.  PMID: 21506964 |
| 34. | [Anti-dermatophyte efficacy and environmental safety of some essential oils commercial and in vitro extracted pure and combined against four keratinophilic pathogenic fungi.](https://pubmed.ncbi.nlm.nih.gov/25953414/)  Ibrahim SY, Abd El-Salam MM.  Environ Health Prev Med. 2015 Jul;20(4):279-86. doi: 10.1007/s12199-015-0462-6. Epub 2015 May 8.  PMID: 25953414 **Free PMC article.** |
| 35. | [Epidemiology of dermatophyte infections among school children in Menoufia Governorate, Egypt.](https://pubmed.ncbi.nlm.nih.gov/29314351/)  Farag AGA, Hammam MA, Ibrahem RA, Mahfouz RZ, Elnaidany NF, Qutubuddin M, Tolba RRE.  Mycoses. 2018 May;61(5):321-325. doi: 10.1111/myc.12743. Epub 2018 Jan 23.  PMID: 29314351 |
| 36. | [Comparative study between topically applied irradiated human amniotic membrane in combination with tea tree oil versus topical tioconazole in pityraisis versicolor treatment.](https://pubmed.ncbi.nlm.nih.gov/32162164/)  Nashwa RK, Ahmed EB, Nemr WA.  Cell Tissue Bank. 2020 Jun;21(2):313-320. doi: 10.1007/s10561-020-09824-5. Epub 2020 Mar 12.  PMID: 32162164 |
| 37. | [Evaluation of long pulsed Nd-YAG laser in the treatment of onychomycosis.](https://pubmed.ncbi.nlm.nih.gov/29768066/)  Ibrahim SA, Albalat W, Ebrahim HM.  J Cosmet Laser Ther. 2019;21(2):76-81. doi: 10.1080/14764172.2018.1469765. Epub 2018 May 16.  PMID: 29768066 No abstract available. |
| 38. | [Public health significance of dermatophytes in Ismailia and Port Said Provinces, Egypt.](https://pubmed.ncbi.nlm.nih.gov/23760076/)  Aboueisha AM, El-Mahallawy H.  Med Mycol J. 2013;54(2):123-9. doi: 10.3314/mmj.54.123.  PMID: 23760076 |
| 39. | [Study of solar photosensitization processes on dermatophytic fungi.](https://pubmed.ncbi.nlm.nih.gov/12916729/)  Ouf SA, Abdel-Kader MH, Shokeir HA, El-Adly AA.  Acta Microbiol Pol. 2003;52(1):65-79.  PMID: 12916729 |
| 40. | [Trichoscopy as a useful method to differentiate tinea capitis from alopecia areata in children at Zagazig University Hospitals.](https://pubmed.ncbi.nlm.nih.gov/27654953/)  Amer M, Helmy A, Amer A.  Int J Dermatol. 2017 Jan;56(1):116-120. doi: 10.1111/ijd.13217. Epub 2016 Sep 22.  PMID: 27654953 |
| 41. | [Efficacy of topical griseofulvin in treatment of tinea corporis.](https://pubmed.ncbi.nlm.nih.gov/16681816/)  Kassem MA, Esmat S, Bendas ER, El-Komy MH.  Mycoses. 2006 May;49(3):232-5. doi: 10.1111/j.1439-0507.2006.01221.x.  PMID: 16681816 Clinical Trial. |
| 42. | [When to suspect tinea; a histopathologic study of 103 cases of PAS-positive tinea.](https://pubmed.ncbi.nlm.nih.gov/27264960/)  Elbendary A, Valdebran M, Gad A, Elston DM.  J Cutan Pathol. 2016 Oct;43(10):852-7. doi: 10.1111/cup.12757. Epub 2016 Jul 12.  PMID: 27264960 |
| 43. | [Fractional CO_2_ laser plus topical antifungal versus fractional CO_2_ laser versus topical antifungal in the treatment of onychomycosis.](https://pubmed.ncbi.nlm.nih.gov/31697010/)  Zaki AM, Abdo HM, Ebadah MA, Ibrahim SM.  Dermatol Ther. 2020 Jan;33(1):e13155. doi: 10.1111/dth.13155. Epub 2019 Nov 14.  PMID: 31697010 Clinical Trial. |
| 44. | [Effect of Different Nail Penetration Enhancers in Solid Lipid Nanoparticles Containing Terbinafine Hydrochloride for Treatment of Onychomycosis.](https://pubmed.ncbi.nlm.nih.gov/33404930/)  Abobakr FE, Fayez SM, Elwazzan VS, Sakran W.  AAPS PharmSciTech. 2021 Jan 6;22(1):33. doi: 10.1208/s12249-020-01893-9.  PMID: 33404930 |
| 45. | [A study of the treatment of cutaneous fungal infection in animal model using photoactivated composite of methylene blue and gold nanoparticle.](https://pubmed.ncbi.nlm.nih.gov/27242275/)  Tawfik AA, Noaman I, El-Elsayyad H, El-Mashad N, Soliman M.  Photodiagnosis Photodyn Ther. 2016 Sep;15:59-69. doi: 10.1016/j.pdpdt.2016.05.010. Epub 2016 May 27.  PMID: 27242275 |
| 46. | [Inhibitory effect of silver nanoparticles mediated by atmospheric pressure air cold plasma jet against dermatophyte fungi.](https://pubmed.ncbi.nlm.nih.gov/26296782/)  Ouf SA, El-Adly AA, Mohamed AH.  J Med Microbiol. 2015 Oct;64(10):1151-1161. doi: 10.1099/jmm.0.000133. Epub 2015 Jul 14.  PMID: 26296782 |
| 47. | [Enhancement of the antidermatophytic activity of silver nanoparticles by Q-switched Nd:YAG laser and monoclonal antibody conjugation.](https://pubmed.ncbi.nlm.nih.gov/28339548/)  Ouf SA, Mohamed AH, El-Adly AA.  Med Mycol. 2017 Jul 1;55(5):495-506. doi: 10.1093/mmy/myw096.  PMID: 28339548 |
| 48. | [Formulation, characterization, and clinical evaluation of microemulsion containing clotrimazole for topical delivery.](https://pubmed.ncbi.nlm.nih.gov/21725708/)  Hashem FM, Shaker DS, Ghorab MK, Nasr M, Ismail A.  AAPS PharmSciTech. 2011 Sep;12(3):879-86. doi: 10.1208/s12249-011-9653-7. Epub 2011 Jul 2.  PMID: 21725708 **Free PMC article.** |
| 49. | [Oil of bitter orange: new topical antifungal agent.](https://pubmed.ncbi.nlm.nih.gov/8737885/)  Ramadan W, Mourad B, Ibrahim S, Sonbol F.  Int J Dermatol. 1996 Jun;35(6):448-9. doi: 10.1111/j.1365-4362.1996.tb03032.x.  PMID: 8737885 Clinical Trial. |
| 50. | [Enhancement of the topical tolnaftate delivery for the treatment of tinea pedis via provesicular gel systems.](https://pubmed.ncbi.nlm.nih.gov/27666873/)  AbouSamra MM, Salama AH.  J Liposome Res. 2017 Dec;27(4):324-334. doi: 10.1080/08982104.2016.1239634. Epub 2016 Oct 19.  PMID: 27666873 |
| 51. | [Onychomycosis in Egypt.](https://pubmed.ncbi.nlm.nih.gov/4249711/)  Abdel-Fattah A, el-Mazny H, Abdullah MA, Refai M.  Mykosen. 1969 Aug 1;12(8):503-6. doi: 10.1111/j.1439-0507.1969.tb03494.x.  PMID: 4249711 No abstract available. |
| 52. | [A comparative clinical and mycological study of Nd-YAG laser versus topical terbinafine in the treatment of onychomycosis.](https://pubmed.ncbi.nlm.nih.gov/25669435/)  El-Tatawy RA, Abd El-Naby NM, El-Hawary EE, Talaat RA.  J Dermatolog Treat. 2015 Oct;26(5):461-4. doi: 10.3109/09546634.2014.998607. Epub 2015 Feb 11.  PMID: 25669435 Clinical Trial. |
| 53. | [Disseminated cryptococcosis with cutaneous lesions.](https://pubmed.ncbi.nlm.nih.gov/8935633/)  Mostafa WZ, Ishak EA, Ekladious EM, Arnaout HH.  J Dermatol. 1996 Mar;23(3):209-13. doi: 10.1111/j.1346-8138.1996.tb03999.x.  PMID: 8935633 |
| 54. | [Importance of mycological confirmation of clinically suspected cases of tinea corporis, tinea pedis and tinea cruris.](https://pubmed.ncbi.nlm.nih.gov/16916049/)  Omar AA.  J Egypt Public Health Assoc. 2004;79(1-2):43-58.  PMID: 16916049 |
| 55. | [Applicability of Fourier transform infrared (FTIR) spectroscopy in rapid identification of some Candida and dermatophyte species infections in humans.](https://pubmed.ncbi.nlm.nih.gov/27337493/)  Mohammed YF, Salem EZ, Shahin IM, Abdo HM, Emam HE, Fawzy M, Abdel Salam MF.  Int J Dermatol. 2016 Oct;55(10):1164-71. doi: 10.1111/ijd.4957. Epub 2016 Jun 23.  PMID: 27337493 |
| 56. | [A multicenter clinicomycological study evaluating the spectrum of adult tinea capitis in Egypt.](https://pubmed.ncbi.nlm.nih.gov/24336943/)  El-Khalawany M, Shaaban D, Hassan H, Abdalsalam F, Eassa B, Abdel Kader A, Shaheen I.  Acta Dermatovenerol Alp Pannonica Adriat. 2013 Dec;22(4):77-82.  PMID: 24336943 |
| 57. | [Basic fibroblast growth factor and tumour necrosis factor alpha in vitiligo and other hypopigmented disorders: suggestive possible therapeutic targets.](https://pubmed.ncbi.nlm.nih.gov/22151832/)  Seif El Nasr H, Shaker OG, Fawzi MM, El-Hanafi G.  J Eur Acad Dermatol Venereol. 2013 Jan;27(1):103-8. doi: 10.1111/j.1468-3083.2011.04368.x. Epub 2011 Dec 8.  PMID: 22151832 |
| 58. | [One-week therapy with oral terbinafine in cases of tinea cruris/corporis.](https://pubmed.ncbi.nlm.nih.gov/7999601/)  Farag A, Taha M, Halim S.  Br J Dermatol. 1994 Nov;131(5):684-6. doi: 10.1111/j.1365-2133.1994.tb04983.x.  PMID: 7999601 Clinical Trial. |
| 59. | [Madurella mycetomi as a cause of maduromycosis in Egypt. Clinical and mycopathological studies of two cases.](https://pubmed.ncbi.nlm.nih.gov/4474964/)  Abdei-Aal H, Karim AZ, Moawad MK, Kalifa TM.  Dermatol Monatsschr. 1974 Aug;160(8):670-4.  PMID: 4474964 No abstract available. |
| 60. | [Single-step PCR using (GACA)4 primer: utility for rapid identification of dermatophyte species and strains.](https://pubmed.ncbi.nlm.nih.gov/18579714/)  Shehata AS, Mukherjee PK, Aboulatta HN, el-Akhras AI, Abbadi SH, Ghannoum MA.  J Clin Microbiol. 2008 Aug;46(8):2641-5. doi: 10.1128/JCM.00697-08. Epub 2008 Jun 25.  PMID: 18579714 **Free PMC article.** |
| 61. | [Could nanovesicles containing a penetration enhancer clinically improve the therapeutic outcome in skin fungal diseases?](https://pubmed.ncbi.nlm.nih.gov/26135513/)  Bsieso EA, Nasr M, Moftah NH, Sammour OA, Abd El Gawad NA.  Nanomedicine (Lond). 2015;10(13):2017-31. doi: 10.2217/nnm.15.49. Epub 2015 Jul 2.  PMID: 26135513 |
| 62. | [Skin granuloma in the Nile Valley.](https://pubmed.ncbi.nlm.nih.gov/5425322/)  el Mofty AM, Nada MM.  Int J Dermatol. 1970 Jan-Mar;9(1):33-40. doi: 10.1111/j.1365-4362.1970.tb04725.x.  PMID: 5425322 No abstract available. |
| 63. | [Epidemiological trends of superficial fungal infections in Upper Egypt: a cohort observational study.](https://pubmed.ncbi.nlm.nih.gov/29952296/)  Saleh R, Abd Elmaged WM, El-Saied DA.  Eur J Dermatol. 2018 Aug 1;28(4):528-530. doi: 10.1684/ejd.2018.3307.  PMID: 29952296 No abstract available. |
| 64. | [Tinea versicolor of the scalp.](https://pubmed.ncbi.nlm.nih.gov/7591430/)  el-Gothamy Z, Ghozzi M.  Int J Dermatol. 1995 Aug;34(8):533-4. doi: 10.1111/j.1365-4362.1995.tb02946.x.  PMID: 7591430 |
| 65. | [Clinical study of a new preparation of 8-methoxypsoralen in photochemotherapy.](https://pubmed.ncbi.nlm.nih.gov/7960360/)  el-Mofty AM, el-Sawalhy H, el-Mofty M.  Int J Dermatol. 1994 Aug;33(8):588-92. doi: 10.1111/j.1365-4362.1994.tb02904.x.  PMID: 7960360 Clinical Trial. |
| 66. | [Coadministration of ketoconazole to cyclosporin-treated kidney transplant recipients: a prospective randomized study.](https://pubmed.ncbi.nlm.nih.gov/8546171/)  Sobh M, el-Agroudy A, Moustafa F, Harras F, el-Bedewy M, Ghoneim M.  Am J Nephrol. 1995;15(6):493-9. doi: 10.1159/000168892.  PMID: 8546171 Clinical Trial. |
| 67. | [Nail dermoscopy is a helpful tool in the diagnosis of onychomycosis: A case control study.](https://pubmed.ncbi.nlm.nih.gov/26576042/)  El-Hoshy KH, Abdel Hay RM, El-Sherif RH, Salah Eldin M, Moussa MF.  Eur J Dermatol. 2015 Sep-Oct;25(5):494-5. doi: 10.1684/ejd.2015.2637.  PMID: 26576042 No abstract available. |
| 68. | [Invasive zygomycosis with a fatal outcome.](https://pubmed.ncbi.nlm.nih.gov/16230557/)  Abdel-Naser MB, Yousef N, el-Fakar NZ, Abdullatif OH, Wollina U, Abdallah MA.  Arch Dermatol. 2005 Oct;141(10):1211-3. doi: 10.1001/archderm.141.10.1211.  PMID: 16230557 No abstract available. |
| 69. | [Animal ringworm in upper Egypt.](https://pubmed.ncbi.nlm.nih.gov/1265571/)  Abou-Gabal M, El-Galil GA, El-Nor EA, El-Rehim DA.  Sabouraudia. 1976 Mar;14(1):33-6. doi: 10.1080/00362177685190061.  PMID: 1265571 |
| 70. | [Laboratory acquired infection with Keratinomyces ajelloi.](https://pubmed.ncbi.nlm.nih.gov/5464089/)  Refai M, Ali AH.  Mykosen. 1970 Jun 1;13(6):317-8. doi: 10.1111/j.1439-0507.1970.tb01277.x.  PMID: 5464089 No abstract available. |
| 71. | [Some deep mycoses prevalent in the United Arab Republic.](https://pubmed.ncbi.nlm.nih.gov/4497480/)  el-Zawahry M.  Int J Dermatol. 1974 Jul-Aug;13(4):210-4. doi: 10.1111/j.1365-4362.1974.tb01795.x.  PMID: 4497480 No abstract available. |
| 72. | [A study of dermatophytoses in Sana'a, Yemen Republic.](https://pubmed.ncbi.nlm.nih.gov/12000511/)  Mahmoud AL.  Mycoses. 2002 Apr;45(3-4):105-8. doi: 10.1046/j.1439-0507.2002.00729.x.  PMID: 12000511 |
| 73. | [Progressive cutaneous Cryptococcosis complicated with meningitis in a myasthenia gravis patient on long-term immunosuppressive therapy - a case report.](https://pubmed.ncbi.nlm.nih.gov/28446137/)  Huong NTC, Altibi AMA, Hoa NM, Tuan LA, Salman S, Morsy S, Lien NTB, Truong NT, Mai NTH, Hoa PTL, Thang NB, Trung VT.  BMC Infect Dis. 2017 Apr 26;17(1):311. doi: 10.1186/s12879-017-2415-8.  PMID: 28446137 **Free PMC article.** |
| 74. | [Chronic mucocutaneous candidiasis associated with an SH2 domain gain-of-function mutation that enhances STAT1 phosphorylation.](https://pubmed.ncbi.nlm.nih.gov/26948078/)  Sobh A, Chou J, Schneider L, Geha RS, Massaad MJ.  J Allergy Clin Immunol. 2016 Jul;138(1):297-299. doi: 10.1016/j.jaci.2015.12.1320. Epub 2016 Mar 2.  PMID: 26948078 No abstract available. |
| 75. | [Identification of three yeast species using the conventional and internal transcribed spacer region sequencing methods as first or second global record from human superficial infections.](https://pubmed.ncbi.nlm.nih.gov/27392537/)  Abdel-Sater MA, Moubasher AA, Soliman Z.  Mycoses. 2016 Oct;59(10):652-61. doi: 10.1111/myc.12520. Epub 2016 Jul 9.  PMID: 27392537 |
| 76. | [Naftifine versus miconazole/hydrocortisone in inflammatory dermatophyte infections.](https://pubmed.ncbi.nlm.nih.gov/7960355/)  Nada M, Hanafi S, al-Omari H, Mokhtar M, el-Shamy S, Mühlbacher J.  Int J Dermatol. 1994 Aug;33(8):570-2. doi: 10.1111/j.1365-4362.1994.tb02899.x.  PMID: 7960355 Clinical Trial. No abstract available. |
| 77. | [Ringworm of the scalp in primary-school children in Alexandria: infection and carriage.](https://pubmed.ncbi.nlm.nih.gov/12197355/)  Omar AA.  East Mediterr Health J. 2000 Sep-Nov;6(5-6):961-7.  PMID: 12197355 |
| 78. | [Incidence of dermatophytes and cyclohexamide resistant fungi on healthy children hairs and nails in nurseries.](https://pubmed.ncbi.nlm.nih.gov/12206316/)  Maghazy SM.  Mycopathologia. 2002;154(4):171-5. doi: 10.1023/a:1016381629089.  PMID: 12206316 |
| 79. | [Topically applied griseofulvin in the treatment of superficial dermatomycoses in Egypt.](https://pubmed.ncbi.nlm.nih.gov/913867/)  Abdel-Aal H, EL-Shazli M, Saleh AM.  J Int Med Res. 1977;5(5):382-6. doi: 10.1177/030006057700500517.  PMID: 913867 |
| 80. | [Trichophyton rubrum infection in a family transmitted from a cat.](https://pubmed.ncbi.nlm.nih.gov/5761179/)  Refai M, Miligy M.  Mykosen. 1968 Mar 1;11(3):191-4. doi: 10.1111/j.1439-0507.1968.tb03320.x.  PMID: 5761179 No abstract available. |
| 81. | [Studies on pityriasis versicolor in Egypt. II. Clinical and therapeutic.](https://pubmed.ncbi.nlm.nih.gov/5110393/)  el-Hefnawi H, el-Gothamy Z, Refai M.  Mykosen. 1971 Oct 1;14(10):483-90. doi: 10.1111/j.1439-0507.1971.tb02936.x.  PMID: 5110393 No abstract available. |
| 82. | [A survey of histoplasmosis and blastomycosis in U.A.R. (Egyptian sector). A preliminary report.](https://pubmed.ncbi.nlm.nih.gov/5796000/)  el-Mofty AM, Mikhail GR, Nada MM, Moawad MK.  Mycopathol Mycol Appl. 1969 May 23;37(3):257-62. doi: 10.1007/BF02051359.  PMID: 5796000 No abstract available. |
| 83. | [The perfect state of Trichophyton violaceum.](https://pubmed.ncbi.nlm.nih.gov/7928033/)  Taha M, Amer M, Salem A, el Harras M.  Int J Dermatol. 1994 Jul;33(7):493-5. doi: 10.1111/j.1365-4362.1994.tb02862.x.  PMID: 7928033 |
| 84. | [[NOTES ON DERMATOSES IN EGYPT].](https://pubmed.ncbi.nlm.nih.gov/14127037/)  ZAWAHRYMM EL.  Maroc Med. 1964 Jan;43:46-53.  PMID: 14127037 French. No abstract available. |
| 85. | [ONYCHOMYCOSIS GENERAL REVIEW AND TREATMENT.](https://pubmed.ncbi.nlm.nih.gov/14162103/)  ELZAWAHRY M.  J Egypt Med Assoc. 1963;46:1267-9.  PMID: 14162103 No abstract available. |
| 86. | [The frequency of causative dermatophytes in Egypt.](https://pubmed.ncbi.nlm.nih.gov/7263125/)  Amer M, Taha M, Tosson Z, El-Garf A.  Int J Dermatol. 1981 Jul-Aug;20(6):431-4. doi: 10.1111/j.1365-4362.1981.tb02009.x.  PMID: 7263125 No abstract available. |
| 87. | [[The enzymatic activity of fungi strains isolated from the skin and skin appendages of humans returning from the tropics].](https://pubmed.ncbi.nlm.nih.gov/16886418/)  Głowacka A, Ochecka-Szymańska A.  Wiad Parazytol. 2001;47(4):729-33.  PMID: 16886418 Polish. |
| 88. | [ERYTHEMA ANNULARE CENTRIFUGUM AS A DERMATOPHYTID.](https://pubmed.ncbi.nlm.nih.gov/14162117/)  ELMOFTY AM, NADA MM.  J Egypt Med Assoc. 1963;46:979-84.  PMID: 14162117 No abstract available. |
| 89. | [Class-specific antibody in human dermatophytosis reactive with Trichophyton rubrum derived antigen.](https://pubmed.ncbi.nlm.nih.gov/7984217/)  Hamouda T, Jeffries CD, Ekladios EM, el-Mishad AM, el-Koomy M, Saleh N.  Mycopathologia. 1994 Aug;127(2):83-8. doi: 10.1007/BF01103063.  PMID: 7984217 |
| 90. | [SPOROTRICHOSIS IN EGYPT.](https://pubmed.ncbi.nlm.nih.gov/14314418/)  EL-MOFTY AM, NADA M.  Br J Dermatol. 1965 Jul;77:357-64. doi: 10.1111/j.1365-2133.1965.tb14661.x.  PMID: 14314418 No abstract available. |
| 91. | [The radiological picture of mycetoma; report of a case verified by biopsy.](https://pubmed.ncbi.nlm.nih.gov/18143687/)  EL DINE MASSOUD G.  J Egypt Med Assoc. 1949 Jan;32(1):64-8.  PMID: 18143687 No abstract available. |
| 92. | [Fluconazole in the treatment of tinea versicolor. Egyptian Fluconazole Study Group.](https://pubmed.ncbi.nlm.nih.gov/9466206/)  Amer MA.  Int J Dermatol. 1997 Dec;36(12):940-2. doi: 10.1046/j.1365-4362.1997.00213.x.  PMID: 9466206 Clinical Trial. No abstract available. |
| 93. | [Tinea capitis in Egypt.](https://pubmed.ncbi.nlm.nih.gov/5595806/)  Abdel Fattah A, El-Gothamy Z.  Mykosen. 1967 May 1;10(5):189-94. doi: 10.1111/j.1439-0507.1967.tb02850.x.  PMID: 5595806 No abstract available. |
| 94. | [Studies on pityriasis versicolor in Egypt. I. Incidence.](https://pubmed.ncbi.nlm.nih.gov/5575840/)  el-Hefnawi H, el-Gothamy Z, Refai M.  Mykosen. 1971 May 1;14(5):225-31. doi: 10.1111/j.1439-0507.1971.tb03041.x.  PMID: 5575840 No abstract available. |
| 95. | [Clinical experience with Canesten-cream (Bay b 5097) against dermatophytes in Egypt.](https://pubmed.ncbi.nlm.nih.gov/4771479/)  Aal HA, Moawad MK.  Mykosen. 1973 Nov 1;16(11):369-71. doi: 10.1111/j.1439-0507.1973.tb04075.x.  PMID: 4771479 No abstract available. |
| 96. | [Primary cutaneous cryptococcosis in Egypt.](https://pubmed.ncbi.nlm.nih.gov/1176246/)  Abdel-Fattah A, Zeid MS, Ghaly AF.  Int J Dermatol. 1975 Oct;14(8):606-9. doi: 10.1111/j.1365-4362.1975.tb00149.x.  PMID: 1176246 |
| 97. | [[Tinea of the scalp in Egypt].](https://pubmed.ncbi.nlm.nih.gov/6872104/)  Othman T, Vacher C.  Bull Soc Pathol Exot Filiales. 1983 Mar-Apr;76(2):126-8.  PMID: 6872104 French. |
| 98. | [[Fungal diseases among the people and animals of Egypt].](https://pubmed.ncbi.nlm.nih.gov/997879/)  Refai M.  Vestn Dermatol Venerol. 1976 Jul;(7):36-40.  PMID: 997879 Russian. No abstract available. |
| 99. | [INCIDENCE OF FUNGUS INFECTION IN U.A.R.](https://pubmed.ncbi.nlm.nih.gov/14080707/)  ZAWAHRYM EL.  Indian J Dermatol. 1963 Oct;9:9-10.  PMID: 14080707 No abstract available. |
| 100. | [NOTES CONCERNING DERMATOSES IN OUR COUNTRIES.](https://pubmed.ncbi.nlm.nih.gov/14162097/)  EL-ZAWAHRY M.  J Egypt Med Assoc. 1963;46:1155-62.  PMID: 14162097 No abstract available. |
| 101. | [TREATMENT OF EPIDERMAL MYCOSES WITH ASTEROL.](https://pubmed.ncbi.nlm.nih.gov/14162112/)  MALEK AI.  J Egypt Med Assoc. 1963;46:1357-60.  PMID: 14162112 No abstract available. |
| 102. | [Incidence of fungus infection in U.A.R.](https://pubmed.ncbi.nlm.nih.gov/13993651/)  EL-ZAWAHRY M.  J Egypt Med Assoc. 1963;46:78-81.  PMID: 13993651 No abstract available. |
| 103. | [Is thallium epilation dangerous in childhood?](https://pubmed.ncbi.nlm.nih.gov/13070299/)  KENEDY DA.  J Egypt Med Assoc. 1953;36(1):59-68.  PMID: 13070299 No abstract available. |
| 104. | [Study of tinea pedis in Egypt.](https://pubmed.ncbi.nlm.nih.gov/13752862/)  KHAFAGY HM.  J Egypt Med Assoc. 1960;43:489-90.  PMID: 13752862 No abstract available. |
| 105. | [Study of tinea cruris in Egypt.](https://pubmed.ncbi.nlm.nih.gov/4636066/)  el-Mazny H, Abdel-Fattah A, Abdallah MA, Refai M.  Mykosen. 1972 Aug 1;15(8):331-5. doi: 10.1111/j.1439-0507.1972.tb02526.x.  PMID: 4636066 No abstract available. |
| 106. | [Studies on pityriasis versicolor in Egypt. 3. Laboratory diagnosis and experimental infection.](https://pubmed.ncbi.nlm.nih.gov/5075545/)  el-Hefnawi H, el-Gothamy Z, Refai M.  Mykosen. 1972 Apr;15(4):165-70. doi: 10.1111/j.1439-0507.1972.tb02468.x.  PMID: 5075545 No abstract available. |
| 107. | [NOTES CONCERNING FUNGUS INFECTION IN U.A.R.](https://pubmed.ncbi.nlm.nih.gov/14162090/)  EL-ZAWAHRY M.  J Egypt Med Assoc. 1963;46:1051-65.  PMID: 14162090 No abstract available. |
| 108. | [Clinical and mycological study of the macerated toe web in Egypt.](https://pubmed.ncbi.nlm.nih.gov/5548033/)  Abd-Allah MA, Abdel-Fattah A, el Mazny, Refai M.  Mykosen. 1971 Feb 1;14(2):83-8. doi: 10.1111/j.1439-0507.1971.tb02993.x.  PMID: 5548033 No abstract available. |
| 109. | [Ringworm in animals in a farm in Assiut.](https://pubmed.ncbi.nlm.nih.gov/5102750/)  Abdallah IS, Gelil GA, Hamid YM, Refai M.  Mykosen. 1971 Apr 1;14(4):175-8. doi: 10.1111/j.1439-0507.1971.tb03024.x.  PMID: 5102750 No abstract available. |
| 110. | [[Contribution to the dermatophyte flora of Northern Egypt].](https://pubmed.ncbi.nlm.nih.gov/5595373/)  Refai M.  Mykosen. 1967 Feb 1;10(2):61-2.  PMID: 5595373 German. No abstract available. |
| 111. | [Oral and cutaneous candidosis in Saudi Arabian newborn infants.](https://pubmed.ncbi.nlm.nih.gov/6948679/)  Sadek S, Mostafa MH, Radwan Z.  Egypt Dent J. 1980 Jan;26(1):73-86.  PMID: 6948679 No abstract available. |
| 112. | [TINEA CAPITIS: A STUDY OF 400 EGYPTIAN CASES.](https://pubmed.ncbi.nlm.nih.gov/14176377/)  TAYLOR WW Jr, BASSALY M.  J Trop Med Hyg. 1964 Jul;67:180-2.  PMID: 14176377 No abstract available. |
| 113. | [A survey of the pathogenic fungi of mycotic infection of the scalp in U.A.R.](https://pubmed.ncbi.nlm.nih.gov/5650626/)  El Mofty AM, Jeffries CD, El Komy HM.  Mycopathol Mycol Appl. 1968 Jan 19;34(1):61-4. doi: 10.1007/BF02050846.  PMID: 5650626 No abstract available. |
| 114. | [Cattie ringworm caused by Trichophyton verrucosum as a source of tinea capitis in children.](https://pubmed.ncbi.nlm.nih.gov/13819849/)  EL-FIKI AY, RIETH H.  J Egypt Med Assoc. 1959;42:540-3.  PMID: 13819849 No abstract available. |
| 115. | [Tinea capitis; a mycological study of three hundred and three cases.](https://pubmed.ncbi.nlm.nih.gov/13184069/)  MIKHAIL GR.  J Egypt Med Assoc. 1954 Jun;37(5):538-54.  PMID: 13184069 No abstract available.   - Search results selected for abstract/title screening   **Search: (((((((((((((Mycetoma[MeSH Major Topic]) OR (Actinomycetoma[MeSH Terms])) OR (Eumycetoma[MeSH Terms])) OR (Madura Foot[MeSH Terms])) OR (Maduromycosis[MeSH Terms])) OR ("actinomycetoma"[All Fields])) OR ("eumycetoma"[All Fields])) OR ("madura foot"[All Fields])) OR ("maduromycosis"[All Fields])) OR ("mycetoma"[All Fields])) AND (Egypt) ("mycetoma"[MeSH Major Topic] OR "mycetoma"[MeSH Terms] OR "mycetoma"[MeSH Terms] OR "mycetoma"[MeSH Terms] OR "mycetoma"[MeSH Terms] OR "actinomycetoma"[All Fields] OR "eumycetoma"[All Fields] OR "madura foot"[All Fields] OR "maduromycosis"[All Fields] OR "mycetoma"[All Fields]) ) OR (Dermatomycoses[MeSH Major Topic])) OR ("dermatomycoses"[All Fields])) AND ("egypt"[All Fields])**  Items 1-29 of 29 ([Display the 29 citations in PubMed](https://pubmed.ncbi.nlm.nih.gov/?term=14314419,4474964,5425322,4497480,18143687,997879,13993651,14162097,14080707,14162090,26483983,14127037,25330098,30946748,26261140,28188051,20849434,32266837,26413063,28522423,21725708,26135513,29952296,8546171,16886418,14162112,14461148,5548033,5650626))   \| 1. \| [MADURA FOOT" IN EGYPT.](https://pubmed.ncbi.nlm.nih.gov/14314419/)  EL-MOFTY AM, ISKANDER IO, NADA M, ZAKI SM.  Br J Dermatol. 1965 Jul;77:365-72. doi: 10.1111/j.1365-2133.1965.tb14662.x.  PMID: 14314419 No abstract available. \| \| --- \| --- \| \| 2. \| [Madurella mycetomi as a cause of maduromycosis in Egypt. Clinical and mycopathological studies of two cases.](https://pubmed.ncbi.nlm.nih.gov/4474964/)  Abdei-Aal H, Karim AZ, Moawad MK, Kalifa TM.  Dermatol Monatsschr. 1974 Aug;160(8):670-4.  PMID: 4474964 No abstract available. \| \| 3. \| [Skin granuloma in the Nile Valley.](https://pubmed.ncbi.nlm.nih.gov/5425322/)  el Mofty AM, Nada MM.  Int J Dermatol. 1970 Jan-Mar;9(1):33-40. doi: 10.1111/j.1365-4362.1970.tb04725.x.  PMID: 5425322 No abstract available. \| \| 4. \| [Some deep mycoses prevalent in the United Arab Republic.](https://pubmed.ncbi.nlm.nih.gov/4497480/)  el-Zawahry M.  Int J Dermatol. 1974 Jul-Aug;13(4):210-4. doi: 10.1111/j.1365-4362.1974.tb01795.x.  PMID: 4497480 No abstract available. \| \| 5. \| [The radiological picture of mycetoma; report of a case verified by biopsy.](https://pubmed.ncbi.nlm.nih.gov/18143687/)  EL DINE MASSOUD G.  J Egypt Med Assoc. 1949 Jan;32(1):64-8.  PMID: 18143687 No abstract available. \| \| 6. \| [[Fungal diseases among the people and animals of Egypt].](https://pubmed.ncbi.nlm.nih.gov/997879/)  Refai M.  Vestn Dermatol Venerol. 1976 Jul;(7):36-40.  PMID: 997879 Russian. No abstract available. \| \| 7. \| [Incidence of fungus infection in U.A.R.](https://pubmed.ncbi.nlm.nih.gov/13993651/)  EL-ZAWAHRY M.  J Egypt Med Assoc. 1963;46:78-81.  PMID: 13993651 No abstract available. \| \| 8. \| [NOTES CONCERNING DERMATOSES IN OUR COUNTRIES.](https://pubmed.ncbi.nlm.nih.gov/14162097/)  EL-ZAWAHRY M.  J Egypt Med Assoc. 1963;46:1155-62.  PMID: 14162097 No abstract available. \| \| 9. \| [INCIDENCE OF FUNGUS INFECTION IN U.A.R.](https://pubmed.ncbi.nlm.nih.gov/14080707/)  ZAWAHRYM EL.  Indian J Dermatol. 1963 Oct;9:9-10.  PMID: 14080707 No abstract available. \| \| 10. \| [NOTES CONCERNING FUNGUS INFECTION IN U.A.R.](https://pubmed.ncbi.nlm.nih.gov/14162090/)  EL-ZAWAHRY M.  J Egypt Med Assoc. 1963;46:1051-65.  PMID: 14162090 No abstract available. \| \| 11. \| [Eumycetoma Osteomyelitis of the Calcaneus in a Child: A Radiologic-Pathologic Correlation following Total Calcanectomy.](https://pubmed.ncbi.nlm.nih.gov/26483983/)  El-Sobky TA, Haleem JF, Samir S.  Case Rep Pathol. 2015;2015:129020. doi: 10.1155/2015/129020. Epub 2015 Sep 21.  PMID: 26483983 **Free PMC article.** \| \| 12. \| [[NOTES ON DERMATOSES IN EGYPT].](https://pubmed.ncbi.nlm.nih.gov/14127037/)  ZAWAHRYMM EL.  Maroc Med. 1964 Jan;43:46-53.  PMID: 14127037 French. No abstract available. \| \| 13. \| [Mapping the potential risk of mycetoma infection in Sudan and South Sudan using ecological niche modeling.](https://pubmed.ncbi.nlm.nih.gov/25330098/)  Samy AM, van de Sande WW, Fahal AH, Peterson AT.  PLoS Negl Trop Dis. 2014 Oct 16;8(10):e3250. doi: 10.1371/journal.pntd.0003250. eCollection 2014 Oct.  PMID: 25330098 **Free PMC article.** \| \| 14. \| [The Role of Interleukin-1 cytokine family (IL-1β, IL-37) and interleukin-12 cytokine family (IL-12, IL-35) in eumycetoma infection pathogenesis.](https://pubmed.ncbi.nlm.nih.gov/30946748/)  Abushouk A, Nasr A, Masuadi E, Allam G, Siddig EE, Fahal AH.  PLoS Negl Trop Dis. 2019 Apr 4;13(4):e0007098. doi: 10.1371/journal.pntd.0007098. eCollection 2019 Apr.  PMID: 30946748 **Free PMC article.** \| \| 15. \| [Recent advances in topical formulation carriers of antifungal agents.](https://pubmed.ncbi.nlm.nih.gov/26261140/)  Bseiso EA, Nasr M, Sammour O, Abd El Gawad NA.  Indian J Dermatol Venereol Leprol. 2015 Sep-Oct;81(5):457-63. doi: 10.4103/0378-6323.162328.  PMID: 26261140 Review. \| \| 16. \| [Incidence and biodiversity of yeasts, dermatophytes and non-dermatophytes in superficial skin infections in Assiut, Egypt.](https://pubmed.ncbi.nlm.nih.gov/28188051/)  Moubasher AH, Abdel-Sater MA, Soliman Z.  J Mycol Med. 2017 Jun;27(2):166-179. doi: 10.1016/j.mycmed.2017.01.005. Epub 2017 Feb 7.  PMID: 28188051 \| \| 17. \| [Skin infections in Egyptian renal transplant recipients.](https://pubmed.ncbi.nlm.nih.gov/20849434/)  Bakr NI, El-Sawy E, Hamdy AF, Bakr MA.  Transpl Infect Dis. 2011 Apr;13(2):131-5. doi: 10.1111/j.1399-3062.2010.00568.x. Epub 2010 Sep 16.  PMID: 20849434 \| \| 18. \| [Oxiconazole nitrate solid lipid nanoparticles: formulation, in-vitro characterization and clinical assessment of an analogous loaded carbopol gel.](https://pubmed.ncbi.nlm.nih.gov/32266837/)  Mahmoud RA, Hussein AK, Nasef GA, Mansour HF.  Drug Dev Ind Pharm. 2020 May;46(5):706-716. doi: 10.1080/03639045.2020.1752707. Epub 2020 Apr 15.  PMID: 32266837 Clinical Trial. \| \| 19. \| [Dermatophytes and other associated fungi in patients attending to some hospitals in Egypt.](https://pubmed.ncbi.nlm.nih.gov/26413063/)  Abd Elmegeed AS, Ouf SA, Moussa TA, Eltahlawi SM.  Braz J Microbiol. 2015 Jul 1;46(3):799-805. doi: 10.1590/S1517-838246320140615. eCollection 2015 Jul-Sep.  PMID: 26413063 **Free PMC article.** \| \| 20. \| [Sertaconazole nitrate loaded nanovesicular systems for targeting skin fungal infection: In-vitro, ex-vivo and in-vivo evaluation.](https://pubmed.ncbi.nlm.nih.gov/28522423/)  Abdellatif MM, Khalil IA, Khalil MAF.  Int J Pharm. 2017 Jul 15;527(1-2):1-11. doi: 10.1016/j.ijpharm.2017.05.029. Epub 2017 May 15.  PMID: 28522423 \| \| 21. \| [Formulation, characterization, and clinical evaluation of microemulsion containing clotrimazole for topical delivery.](https://pubmed.ncbi.nlm.nih.gov/21725708/)  Hashem FM, Shaker DS, Ghorab MK, Nasr M, Ismail A.  AAPS PharmSciTech. 2011 Sep;12(3):879-86. doi: 10.1208/s12249-011-9653-7. Epub 2011 Jul 2.  PMID: 21725708 **Free PMC article.** \| \| 22. \| [Could nanovesicles containing a penetration enhancer clinically improve the therapeutic outcome in skin fungal diseases?](https://pubmed.ncbi.nlm.nih.gov/26135513/)  Bsieso EA, Nasr M, Moftah NH, Sammour OA, Abd El Gawad NA.  Nanomedicine (Lond). 2015;10(13):2017-31. doi: 10.2217/nnm.15.49. Epub 2015 Jul 2.  PMID: 26135513 \| \| 23. \| [Epidemiological trends of superficial fungal infections in Upper Egypt: a cohort observational study.](https://pubmed.ncbi.nlm.nih.gov/29952296/)  Saleh R, Abd Elmaged WM, El-Saied DA.  Eur J Dermatol. 2018 Aug 1;28(4):528-530. doi: 10.1684/ejd.2018.3307.  PMID: 29952296 No abstract available. \| \| 24. \| [Coadministration of ketoconazole to cyclosporin-treated kidney transplant recipients: a prospective randomized study.](https://pubmed.ncbi.nlm.nih.gov/8546171/)  Sobh M, el-Agroudy A, Moustafa F, Harras F, el-Bedewy M, Ghoneim M.  Am J Nephrol. 1995;15(6):493-9. doi: 10.1159/000168892.  PMID: 8546171 Clinical Trial. \| \| 25. \| [[The enzymatic activity of fungi strains isolated from the skin and skin appendages of humans returning from the tropics].](https://pubmed.ncbi.nlm.nih.gov/16886418/)  Głowacka A, Ochecka-Szymańska A.  Wiad Parazytol. 2001;47(4):729-33.  PMID: 16886418 Polish. \| \| 26. \| [TREATMENT OF EPIDERMAL MYCOSES WITH ASTEROL.](https://pubmed.ncbi.nlm.nih.gov/14162112/)  MALEK AI.  J Egypt Med Assoc. 1963;46:1357-60.  PMID: 14162112 No abstract available. \| \| 27. \| [Treatment of fungus infections with griseofulvin.](https://pubmed.ncbi.nlm.nih.gov/14461148/)  EL-MOFTY AM.  J Egypt Med Assoc. 1961;44:772-87.  PMID: 14461148 No abstract available. \| \| 28. \| [Clinical and mycological study of the macerated toe web in Egypt.](https://pubmed.ncbi.nlm.nih.gov/5548033/)  Abd-Allah MA, Abdel-Fattah A, el Mazny, Refai M.  Mykosen. 1971 Feb 1;14(2):83-8. doi: 10.1111/j.1439-0507.1971.tb02993.x.  PMID: 5548033 No abstract available. \| \| 29. \| [A survey of the pathogenic fungi of mycotic infection of the scalp in U.A.R.](https://pubmed.ncbi.nlm.nih.gov/5650626/)  El Mofty AM, Jeffries CD, El Komy HM.  Mycopathol Mycol Appl. 1968 Jan 19;34(1):61-4. doi: 10.1007/BF02050846.  PMID: 5650626 No abstract available. \|  - Search results selected for assessment of eligibility   **Search: (((((((((((((Mycetoma[MeSH Major Topic]) OR (Actinomycetoma[MeSH Terms])) OR (Eumycetoma[MeSH Terms])) OR (Madura Foot[MeSH Terms])) OR (Maduromycosis[MeSH Terms])) OR ("actinomycetoma"[All Fields])) OR ("eumycetoma"[All Fields])) OR ("madura foot"[All Fields])) OR ("maduromycosis"[All Fields])) OR ("mycetoma"[All Fields])) AND (Egypt) ("mycetoma"[MeSH Major Topic] OR "mycetoma"[MeSH Terms] OR "mycetoma"[MeSH Terms] OR "mycetoma"[MeSH Terms] OR "mycetoma"[MeSH Terms] OR "actinomycetoma"[All Fields] OR "eumycetoma"[All Fields] OR "madura foot"[All Fields] OR "maduromycosis"[All Fields] OR "mycetoma"[All Fields]) ) OR (Dermatomycoses[MeSH Major Topic])) OR ("dermatomycoses"[All Fields])) AND ("egypt"[All Fields])** |

Items 1-12 of 12 ([Display the 12 citations in PubMed](https://pubmed.ncbi.nlm.nih.gov/?term=14314419,4474964,5425322,4497480,18143687,997879,13993651,14162097,14080707,14162090,26483983,14127037))

| 1. | [*MADURA FOOT" IN EGYPT.*](https://pubmed.ncbi.nlm.nih.gov/14314419/)  *EL-MOFTY AM, ISKANDER IO, NADA M, ZAKI SM.*  *Br J Dermatol. 1965 Jul;77:365-72. doi: 10.1111/j.1365-2133.1965.tb14662.x.*  *PMID: 14314419 No abstract available.* |
| --- | --- |
| 2. | [*Madurella mycetomi as a cause of maduromycosis in Egypt. Clinical and mycopathological studies of two cases.*](https://pubmed.ncbi.nlm.nih.gov/4474964/)  *Abdei-Aal H, Karim AZ, Moawad MK, Kalifa TM.*  *Dermatol Monatsschr. 1974 Aug;160(8):670-4.*  *PMID: 4474964 No abstract available.* |
| 3. | [Skin granuloma in the Nile Valley.](https://pubmed.ncbi.nlm.nih.gov/5425322/)  el Mofty AM, Nada MM.  Int J Dermatol. 1970 Jan-Mar;9(1):33-40. doi: 10.1111/j.1365-4362.1970.tb04725.x.  PMID: 5425322 No abstract available. |
| 4. | [Some deep mycoses prevalent in the United Arab Republic.](https://pubmed.ncbi.nlm.nih.gov/4497480/)  el-Zawahry M.  Int J Dermatol. 1974 Jul-Aug;13(4):210-4. doi: 10.1111/j.1365-4362.1974.tb01795.x.  PMID: 4497480 No abstract available. |
| 5. | [*The radiological picture of mycetoma; report of a case verified by biopsy.*](https://pubmed.ncbi.nlm.nih.gov/18143687/)  *EL DINE MASSOUD G.*  *J Egypt Med Assoc. 1949 Jan;32(1):64-8.*  *PMID: 18143687 No abstract available.* |
| 6. | [[Fungal diseases among the people and animals of Egypt].](https://pubmed.ncbi.nlm.nih.gov/997879/)  Refai M.  Vestn Dermatol Venerol. 1976 Jul;(7):36-40.  PMID: 997879 Russian. No abstract available. |
| 7. | [*Incidence of fungus infection in U.A.R.*](https://pubmed.ncbi.nlm.nih.gov/13993651/)  *EL-ZAWAHRY M.*  *J Egypt Med Assoc. 1963;46:78-81.*  *PMID: 13993651 No abstract available.* |
| 8. | [NOTES CONCERNING DERMATOSES IN OUR COUNTRIES.](https://pubmed.ncbi.nlm.nih.gov/14162097/)  EL-ZAWAHRY M.  J Egypt Med Assoc. 1963;46:1155-62.  PMID: 14162097 No abstract available. |
| 9. | [INCIDENCE OF FUNGUS INFECTION IN U.A.R.](https://pubmed.ncbi.nlm.nih.gov/14080707/)  ZAWAHRYM EL.  Indian J Dermatol. 1963 Oct;9:9-10.  PMID: 14080707 No abstract available. |
| 10. | [NOTES CONCERNING FUNGUS INFECTION IN U.A.R.](https://pubmed.ncbi.nlm.nih.gov/14162090/)  EL-ZAWAHRY M.  J Egypt Med Assoc. 1963;46:1051-65.  PMID: 14162090 No abstract available. |
| 11. | [Eumycetoma Osteomyelitis of the Calcaneus in a Child: A Radiologic-Pathologic Correlation following Total Calcanectomy.](https://pubmed.ncbi.nlm.nih.gov/26483983/)  El-Sobky TA, Haleem JF, Samir S.  Case Rep Pathol. 2015;2015:129020. doi: 10.1155/2015/129020. Epub 2015 Sep 21.  PMID: 26483983 **Free PMC article.** |
| 12. | [[NOTES ON DERMATOSES IN EGYPT].](https://pubmed.ncbi.nlm.nih.gov/14127037/)  ZAWAHRYMM EL.  Maroc Med. 1964 Jan;43:46-53.  PMID: 14127037 French. No abstract available. |

- Additional results from a separate search

Item 1-1 of 1 ([Display the 1 citation in PubMed](https://pubmed.ncbi.nlm.nih.gov/?term=21696990))

| 1. | [Clinicopathological features and the practice of diagnosing infectious cutaneous granulomas in Egypt.](https://pubmed.ncbi.nlm.nih.gov/21696990/)  El-Khalawany M, Meraag I, Eassa B, El-Naby HH.  Int J Infect Dis. 2011 Sep;15(9):e620-6. doi: 10.1016/j.ijid.2011.04.014. Epub 2011 Jun 21.  PMID: 21696990 |
| --- | --- |

- **Search results: Final included articles**

| 1. | [*MADURA FOOT" IN EGYPT.*](https://pubmed.ncbi.nlm.nih.gov/14314419/)  *EL-MOFTY AM, ISKANDER IO, NADA M, ZAKI SM.*  *Br J Dermatol. 1965 Jul;77:365-72. doi: 10.1111/j.1365-2133.1965.tb14662.x.*  *PMID: 14314419 No abstract available.* |
| --- | --- |
| 2. | [*Madurella mycetomi as a cause of maduromycosis in Egypt. Clinical and mycopathological studies of two cases.*](https://pubmed.ncbi.nlm.nih.gov/4474964/)  *Abdei-Aal H, Karim AZ, Moawad MK, Kalifa TM.*  *Dermatol Monatsschr. 1974 Aug;160(8):670-4.*  *PMID: 4474964 No abstract available.* |
| 3. | [Skin granuloma in the Nile Valley.](https://pubmed.ncbi.nlm.nih.gov/5425322/)  el Mofty AM, Nada MM.  Int J Dermatol. 1970 Jan-Mar;9(1):33-40. doi: 10.1111/j.1365-4362.1970.tb04725.x.  PMID: 5425322 No abstract available. |
| 4. | [*The radiological picture of mycetoma; report of a case verified by biopsy.*](https://pubmed.ncbi.nlm.nih.gov/18143687/)  *EL DINE MASSOUD G.*  *J Egypt Med Assoc. 1949 Jan;32(1):64-8.*  *PMID: 18143687 No abstract available.* |
| 5. | [*Incidence of fungus infection in U.A.R.*](https://pubmed.ncbi.nlm.nih.gov/13993651/)  *EL-ZAWAHRY M.*  *J Egypt Med Assoc. 1963;46:78-81.*  *PMID: 13993651 No abstract available.* |
| 6. | [Eumycetoma Osteomyelitis of the Calcaneus in a Child: A Radiologic-Pathologic Correlation following Total Calcanectomy.](https://pubmed.ncbi.nlm.nih.gov/26483983/)  El-Sobky TA, Haleem JF, Samir S.  Case Rep Pathol. 2015;2015:129020. doi: 10.1155/2015/129020. Epub 2015 Sep 21.  PMID: 26483983 **Free PMC article.** |
| 7. | [Clinicopathological features and the practice of diagnosing infectious cutaneous granulomas in Egypt.](https://pubmed.ncbi.nlm.nih.gov/21696990/)  El-Khalawany M, Meraag I, Eassa B, El-Naby HH.  Int J Infect Dis. 2011 Sep;15(9):e620-6. doi: 10.1016/j.ijid.2011.04.014. Epub 2011 Jun 21.  PMID: 21696990 |
| 8. | Taha M. Is_mycetoma_endemic_in_Egypt. Available at <https://www.researchgate.net/publication/261708805_Is_mycetoma_endemic_in_Egypt> [Accessed on 24 September 2021] |

**WEB RESOURCES**

World Health Organization (WHO), Mycetoma, <https://www.who.int/news-room/fact-sheets/detail/mycetoma>

Mycetoma Research Centre (MRC), <http://www.mycetoma.edu.sd/>

Drugs for Neglected Diseases initiative (DNDi), <https://dndi.org/diseases/mycetoma/>

Leading International Fungal Infection (LIFE), Mycetoma, <http://life-worldwide.org/mycetoma>

International Society for Human and Animal Mycology (ISHAM), <https://www.isham.org/>
